# Where the minor things are: a pan-eukaryotic survey suggests neutral processes may explain much of minor intron evolution

**DOI:** 10.1093/nar/gkad797

**Published:** 2023-10-11

**Authors:** Graham E Larue, Scott W Roy

**Affiliations:** Quantitative and Systems Biology Graduate Program, University of California Merced, Merced, CA 95343, USA; Department of Molecular and Cell Biology, University of California Merced, Merced, CA 95343, USA; Department of Biology, San Francisco State University, San Francisco, CA 94132, USA

## Abstract

Spliceosomal introns are gene segments removed from RNA transcripts by ribonucleoprotein machineries called spliceosomes. In some eukaryotes a second ‘minor’ spliceosome is responsible for processing a tiny minority of introns. Despite its seemingly modest role, minor splicing has persisted for roughly 1.5 billion years of eukaryotic evolution. Identifying minor introns in over 3000 eukaryotic genomes, we report diverse evolutionary histories including surprisingly high numbers in some fungi and green algae, repeated loss, as well as general biases in their positional and genic distributions. We estimate that ancestral minor intron densities were comparable to those of vertebrates, suggesting a trend of long-term stasis. Finally, three findings suggest a major role for neutral processes in minor intron evolution. First, highly similar patterns of minor and major intron evolution contrast with both functionalist and deleterious model predictions. Second, observed functional biases among minor intron-containing genes are largely explained by these genes’ greater ages. Third, no association of intron splicing with cell proliferation in a minor intron-rich fungus suggests that regulatory roles are lineage-specific and thus cannot offer a general explanation for minor splicing’s persistence. These data constitute the most comprehensive view of minor introns and their evolutionary history to date, and provide a foundation for future studies of these remarkable genetic elements.

## Graphical Abstract

**Graphical Abstract:**
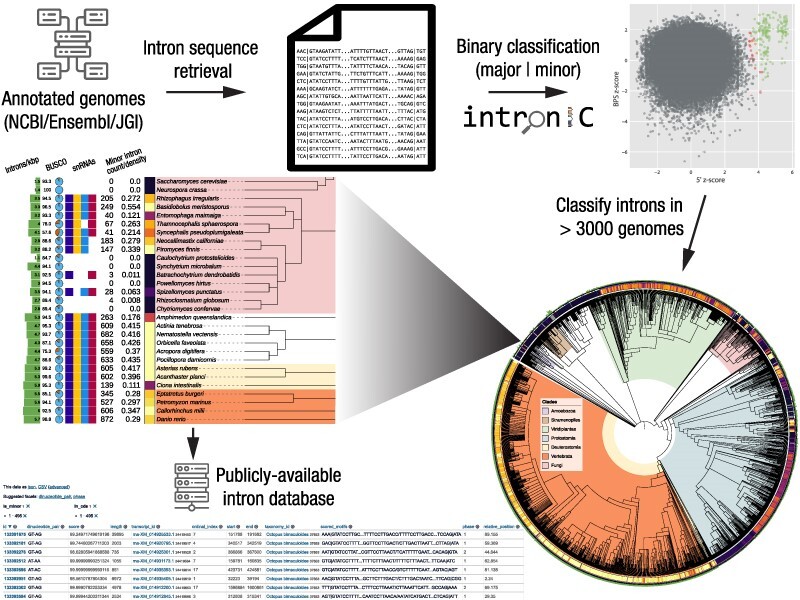

## Introduction

Spliceosomal introns are sequences in eukaryotic genes that are removed (spliced) from the pre-mRNA transcripts of genes by machinery known as the spliceosome prior to maturation and nuclear export of the final mRNA (1–3). For the better part of a decade after spliceosomal introns (hereafter simply introns) were first characterized in eukaryotic genomes (4–8), it was assumed that all introns shared a fixed set of consensus dinucleotide termini—GT at the beginning (5′ side) and AG at the end (3′ side)—and were processed by the same core machinery (9,10). This view was revised after the discovery of a small number of introns with AT-AC termini (10,11); shortly thereafter, an entirely separate spliceosome was described that could process these aberrant introns (12,13), termed the U12-dependent or minor spliceosome. The minor spliceosome, like its counterpart now known as the major/U2-dependent spliceosome, has origins early in eukaryotic evolution (14). While initially documented as having only AT-AC termini, it has since been shown that the majority of minor introns in most species are in fact of the GT-AG subtype, although introns with an increasing diversity of termini (so-called ‘non-canonical’ introns, with boundaries that are not GT-AG, GC-AG or AT-AC) seem to be able to be processed in certain contexts and to varying degrees by both spliceosomes (15–19). Until very recently (20), in every genome investigated minor introns have been found to comprise only a tiny fraction (≲0.5%) of the total set of introns; despite this, they have also been found to be well-conserved over hundreds of millions of years of evolution (21,22) (e.g., 94% of minor introns in human are conserved in chicken; 91% in zebrafish (21)).

While minor introns were almost certainly present in early eukaryotes (14) and are retained in a wide variety of extant eukaryotic species (19,23), to date only two lineages—animals and plants—are known to contain more than a few dozen minor introns(19). Interestingly, in contrast to the massive minor intron loss observed in many lineages, minor intron complements in certain clades are remarkably evolutionarily stable. This contrasting pattern of retention versus massive loss raises a puzzle of minor spliceosomal intron function: if minor introns are not functionally important why are they almost entirely conserved over hundreds of millions of years in some lineages; yet if they are important, how can they be repeatedly decimated or lost entirely in other lineages?

Two observations are particularly relevant to the question of minor spliceosomal intron function. First, over the past ten or so years, some studies have proposed roles for minor introns in cellular differentiation, with decreased minor splicing activity driving downregulation of minor intron-containing genes (MIGs) associated with cessation of cell cycling (24–26). Most compellingly, a recent study showed that the splicing regulator SR10 is regulated at the level of minor splicing, with inefficient splicing leading to downregulation of other SR proteins whose pro-splicing activities promote cell cycle progression (27). Interestingly, a negative association of minor splicing with cell differentiation has also been argued for in plants (24,28). This pattern is curious, given that the common ancestor of animals and plants is thought to have been unicellular and thus not to have undergone terminal differentiation (although recent findings of multicellular stages as well as differentiation-like processes across diverse eukaryotes may ultimately call this common assumption into question (29,30)).

Second, minor introns have been reported to show functional biases, being disproportionately found in genes encoding various core cellular functions including DNA repair, RNA processing, transcription and cell cycle functions that largely appear to hold between plants and animals (24,31) (though the strength of these associations is questioned somewhat in (32)). Particularly given the above evidence that regulation of minor splicing regulates core cellular processes, these patterns would seem to be consistent with an ancient role for minor splicing in cell cycle regulation, that could have been secondarily recruited for multicellular differentiation separately in animals and plants. However, other explanations remain possible. What is needed to understand the evolutionary history and importance of minor spliceosomal introns is genomic and regulatory characterization of additional lineages with relatively large complements of minor spliceosomal introns.

Because minor introns possess sequence motifs distinct from major introns (31,33), it is possible to identify them using sequence-based bioinformatic methods (19,22,31,34–36). Previous studies have cataloged the presence/absence of minor introns/spliceosome components across multiple eukaryotic genomes using various custom tools (22,23,34,35), but many of these studies were necessarily limited by the data available at the time, and by the lack of a published or otherwise convenient computational method to identify minor introns.

In this work, with the substantially larger and more diverse genomic datasets now publicly accessible coupled with the intron classification program intronIC (19), we have been able to aggregate minor intron presence/absence data with higher fidelity than earlier works across a much larger sample of eukaryotic species. Intron metadata for all species identified in this study as containing minor introns is available for querying and download via an online database at https://www.introns.info. By compiling a catalog of minor introns across thousands of eukaryotic species, we provide an unprecedentedly general portrait of minor intron characteristics, diversity, and distribution, and test several important hypotheses about minor intron evolution and function. In addition, we use the discovery of hundreds of minor spliceosomal introns in a mycorrhizal fungus to characterize several features of the minor spliceosomal system across cell types, and suggest that the functional biases long observed in minor introns may largely be explained by the age bias of their parent genes.

## Materials and methods

### Data acquisition

Genomes and annotations for 3107 eukaryotic species were downloaded from the online databases hosted by NCBI (RefSeq and GenBank), JGI and Ensembl using custom Python scripts, and taxonomic information for each species was retrieved from the NCBI Taxonomy Database (37) using the taxadb Python library (https://github.com/HadrienG/taxadb). We used NCBI as our primary resource, since it contains the largest number of species and in many cases serves as the upstream source for a number of other genome resources. Additional species available only from JGI and Ensembl were included for completeness, as were GenBank genomes with available standard annotation files (GTF or GFF; species with only GBFF annotations were excluded). GenBank annotations in particular are of highly variable quality and may be preliminary, draft or otherwise incomplete, which is one of the reasons we chose to also include annotation BUSCO scores for all species. Accession numbers (where available) and retrieval dates of the data for each species are available on FigShare under the following DOI: https://doi.org/10.6084/m9.figshare.20483655.

### Identification of spliceosomal snRNAs

Each genome was searched for the presence of the minor snRNAs U11, U12, U4atac and U6atac using INFERNAL v1.3.3 (38) with covariance models retrieved from Rfam (RF00548, RF00007, RF00618, RF00619). The default E-value cutoff of 0.01 was used to identify positive snRNA matches, and any snRNA with at least one match below the *E*-value cutoff was considered present.

### Classification of minor introns

For every genome with annotated introns, intronIC v1.2.3 (19) was used to identify putative minor introns using default settings, including introns defined by exon features (e.g. introns in UTRs) and excluding any introns shorter than 30 nt. A minor intron score threshold of 90% (the default) was used for all species except *Physarum polycephalum*, where we set the threshold at 95% per (20). Although our substrate data includes UTR introns—at least some of which appear to be involved in the regulation of gene expression (39–43)—the various analyses performed in this study (beyond the simple reporting of per-genome numbers of introns of each type and the intron position analysis) include only introns in protein-coding regions of genes. UTR introns generally are an understudied group of introns, and almost nothing is known about minor introns in UTRs; exploring this in more detail would certainly be an exciting avenue for future research.

### Identification of orthologous introns

A set of custom software was used to identify orthologs between various species as described previously (20). Briefly, transcriptomes for each species in a group were generated using the longest isoforms of each gene (https://github.com/glarue/cdseq). Then, the program reciprologs (https://github.com/glarue/reciprologs) was used in conjunction with DIAMOND v2.0.13 (non-default arguments: - -very-sensitive - -evalue 1e-10) to identify reciprocal best hits (RBHs) between all pairs of species, and to construct an undirected graph using the RBHs as edges. The maximal cliques of such a graph represent orthologous clusters where all members are RBHs of one another. In certain cases where only orthologous MIGs (as opposed to all orthologs) were required, reciprologs was run as part of a pipeline using the –subset argument in combination with separately generated lists of MIGs for each species, which dramatically decreases runtime by constraining the query lists to only include MIGs (producing identical results to the MIG-containing subset of a full alignment). Full ortholog searches were required for all ancestral density reconstructions as well as all comparisons of minor and major intron conservation/loss (e.g., Figure 3A).

Groups of orthologous genes were aligned at the protein level using a combination of MAFFT v7.453 and Clustal Omega v1.2.4. Intron positions were mapped to the amino acid sequences within the alignments, and their conservation states and putative types were collated using a custom Python pipeline (following the approach in (44)). Local ungapped alignment quality of ≥ 40% conserved amino acid identity over a window of 10 residues both upstream and downstream of each intron position was required. Introns in the same position within these orthologous alignments were considered conserved as the same type or as putative instances of type conversion, depending on their minor intron scores. For the analyses of putative intron type conversions (e.g. minor-to-major), major introns were required to have scores ≤ 60% instead of the default threshold of ≤ 90% to minimize the erroneous inclusion of minor introns with borderline scores as major-type, and intron alignments containing introns with such borderline scores (a tiny fraction of the total alignments) were excluded. Intron sliding (the shifting of an individual intron’s boundaries within a gene versus its ancestral location) (45) is not explicitly accounted for by our pipeline (an intron sliding event would be categorized in this context as intron loss in the containing gene); however, this phenomenon is at most very rare and unlikely to meaningfully affect our results (45–48).

### Intron positions within transcripts and intron phase

Information about the relative position of each intron within its parent transcript (represented as a percentage of the total length of the transcript sequence) as well as intron phase (for introns defined by CDS features) is included in the output of intronIC (19). These data were extracted for every species and used in the associated analyses.

### Non-canonical minor introns

The genomes of all species were first analyzed to assess the number of putative non-canonical minor introns they contained, and those with the highest numbers of non-canonical minor introns were used to perform multiple protein-level alignments of orthologous gene sets between different pairs of species. From these alignments, all orthologous intron pairs with a minor intron (minor:minor or minor:major) were collected, and used to build orthologous intron clusters (technically, subgraphs). For animals, human was included in a majority of the alignments to facilitate the generation of larger clusters (where the same intron shared between different pairwise alignments served to group the alignments together); for plants, *Elaeis guineensis* filled the same role. For the analysis of non-canonical intron boundaries in each type of intron (major and minor), homologous clusters were filtered to include only the subset of clusters where at least two introns of the associated type were found; this was done to reduce the likelihood of inclusion of potential false-positive minor introns (e.g., a putative minor intron in an alignment where every other orthologous intron in the cluster is major-type). In addition, a more stringent score threshold of < 60% minor-type probability (rather than ≤ 90%) was used for the identification of major introns in orthologous intron clusters.

### Annotation quality assessment using BUSCO

**Figure 1.**
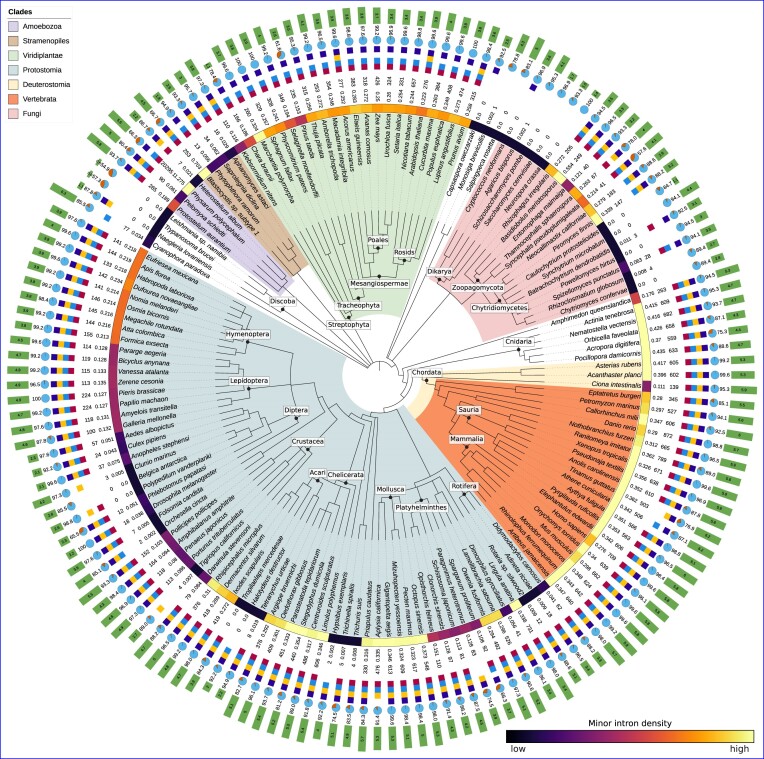
Minor intron densities and other metadata for selected species of interest. The colored strip following the species name represents the relative minor intron density (darker = lower, lighter = higher). Additional data from inside to outside are as follows: minor intron density (%), number of putative minor introns (including introns in non-coding regions of genes), minor snRNAs present in the annotated transcriptome (red: U11, light blue: U12, yellow: U4atac, purple: U6atac), BUSCO score versus the eukaryotic BUSCO gene set, median total (minor and major) intron density in introns/kbp coding sequence. Taxonomic relationships based upon data from the NCBI Taxonomy Database (37); figure generated using iTOL (68). A similar visualization for all species in our dataset can be found in Supplementary Figure S1.

Translated versions of the transcriptomes of all species were searched for broadly-conserved eukaryotic genes using BUSCO v5.3.2 (49) (lineage eukaryota_odb10). Complete BUSCO scores were then integrated into the overall dataset (e.g., Figure 1 and Supplementary Figure S1).

### Curation of minor intron data/edge cases

Given the size of the data involved in our analyses, there are likely to be some number of introns that appear superficially similar to minor introns simply by chance, and because intronIC does not account for additional factors like local context, presence/absence of snRNAs, etc., these introns will be identified as minor-type. In general this is not an issue, as the number of false-positive minor introns per genome is usually very small. However, when summarizing aggregate eukaryotic data and attempting to provide a resource to be used as a reference, some amount of curation is warranted to avoid the inclusion of obviously spurious or misleading results.

To that end, a number of heuristics were used in deciding whether to designate a given species as either confidently containing—or confidently not containing—minor introns. First, it is important to note that intronIC will automatically try to adjust intron boundaries by a short distance if the annotated boundaries are non-canonical and there is a strong minor 5′SS motif within ≈10 bp of the annotated 5′SS. In some poorly-annotated species, or species with otherwise aberrant intron motifs this can lead to increased false positives in the form of putatively minor introns with ‘corrected’ splice boundaries. Such introns are documented by intronIC, and it is therefore possible to determine their proportion in the final number of minor introns reported. The criteria for presence of minor introns in a genome in our dataset is a corrected minor intron fraction of ≤ 0.25, ≥ 3 called minor introns and at least two minor snRNAs.

The criteria for absence of minor introns (assigned the color black in the minor intron density color strip in Figures 1 and S1) is either of the following: ≤ 3 identified minor introns and fewer than two minor snRNAs; ≤ 5 identified minor introns, fewer than two minor snRNAs, fewer than five uncorrected AT-AC minor introns and either RefSeq-based annotations or a BUSCO score greater than or equal to *B*_*Q*1_ − (1.5 × *B*_*IQR*_), where *B*_*Q*1_ is the first quartile of the BUSCO scores of the broad RefSeq category to which the species belongs (i.e., ‘vertebrates’, ‘invertebrates’, ‘plants’, ‘protozoa’, ‘fungi’) and *B*_*IQR*_ is the inner quartile range of such scores. The idea behind this metric is to only assign confident minor intron loss to species whose BUSCO scores aren’t extremely low; very low BUSCO scores could indicate real gene loss or incomplete annotations, and neither of those scenarios forecloses on the possibility that the species may have minor introns (whereas a species with a high BUSCO score and a very low number of minor introns/minor snRNAs is more likely to be genuinely lacking either/both). Finally, species with very low numbers of minor introns and minor snRNAs but very high minor intron densities (≥ 1%) were categorized as uncertain to account for a small number of edge cases with massive intron loss and spurious false positives that, due to the low number of total introns, misleadingly appear to be cases of outstandingly high minor intron density (e.g. *Leishmania martiniquensis*). Importantly, Figure 1 and Supplementary Figure S1 still includes the raw values for each species matching the above criteria; in such cases, however, the minor intron density color strip is gray and the number of reported minor introns is followed by an asterisk (*).

### Calculation of summary statistics (genic intron density, transcript length, etc.)

Transcriptomes for all species were generated using a custom Python script (https://github.com/glarue/cdseq). Each annotated transcript’s length was calculated as the sum of its constituent CDS (coding sequence) features, and the longest isoform was selected for each gene. The number of introns per transcript was computed based on the same CDS data, and combined with the transcript length data to calculate introns/kbp coding sequence (genic intron density) for each gene. Intron lengths were extracted directly from intronIC output, as was intron phase and intron position as a fraction of transcript length. The relative intron position, taken as the point position in the coding sequence where the intron occurs, was calculated as the cumulative sum of the preceding coding sequence divided by the total length of coding sequence in the transcript. For comparisons of intron densities and gene lengths of MIGs and non-MIGs, species with fewer than ten putative minor introns were excluded to avoid inclusion of spurious minor intron calls (inclusion of such introns does not meaningfully change the findings as presented).

### Gene age assignment

To explore the hypothesis that gene age biases might explain differences in average genic intron density and gene length between major and minor introns (Figure 7), GenEra v1.1.1 (50–52) was used to assign age categories to annotated genes in *Homo sapiens*, *Arabidopsis thaliana* and *Basidiobolus meristosporus*. Within the resulting gene age categories for each species, the median lengths and genic intron densities of MIGs and non-MIGs were compared (Supplementary Figure S3), and differences were evaluated with Mann–Whitney *U* tests followed by Benjamini–Hochberg correction.

### Ancestral intron density reconstruction

Reconstructions of ancestral intron complements in different nodes was performed as described in (53). Briefly, for a set of three species α, β and γ where γ is an outgroup to α and β (i.e. α and β are sister with respect to γ), introns shared between any pair of species are (under the assumption of negligible parallel intron gain) *a priori* part of the set of introns in the ancestor of α and β. For all introns shared between a given species pair, for example α and γ (but not necessarily β) *N*_αγ_, the probability of an intron from that set being found in β (in other words, the fraction of ancestral introns retained in β) is


\begin{eqnarray*} \hat{P}_\beta = {N_{\alpha \beta \gamma } \over N_{\alpha \gamma }}, \end{eqnarray*}

where *N*_αβγ_ is the number of introns shared between all three species. Deriving these fractions of ancestral introns for each of the aligned species, $N_\Omega$ is then defined as the total number of ancestral introns in the aligned regions, and its relationship to the conservation states of introns in the alignments of the three species is


\begin{eqnarray*} N_{\alpha \beta \gamma } = N_\Omega ( \hat{P}_\alpha \cdot \hat{P}_\beta \cdot \hat{P}_\gamma ), \end{eqnarray*}

the product of the ancestral intron number and the fraction of ancestral introns present in each species. Finally, solving for the number of ancestral introns produces the estimate


\begin{eqnarray*} \hat{N}_\Omega = \frac{{N_{\alpha \beta } \cdot N_{\alpha \gamma } \cdot N_{\beta \gamma }}}{( N_{\alpha \beta \gamma } )^2}. \end{eqnarray*}

Performing the above procedure for both major and minor introns in a given alignment allows for the estimation of the ancestral minor intron density in the corresponding node as


\begin{eqnarray*} \hat{\rho }_{minor}=\frac{\hat{N}_{\Omega _{minor}}}{\hat{N}_{\Omega _{minor}} + \hat{N}_{\Omega _{major}}} \cdot 100\%. \end{eqnarray*}

Without a point of reference, however, $\hat{\rho }_{minor}$ is difficult to interpret, as the genes included in the alignments are not an especially well-defined set; because they consist of all of the orthologs found between a given trio of species, their composition is likely to change at least somewhat for each unique group of aligned species representing the same ancestral node. We addressed this issue by normalizing $\hat{\rho }_{minor}$ to the minor intron density of a chosen reference species included in each group. For example, in our reconstructions of ancestral intron densities in animals, we included human in every distinct set of species as either an ingroup or outgroup member. After calculating the estimated minor intron density of a given ancestral node, we then divided that value by the minor intron density of the human genes present in the same alignments to produce the estimated ancestral minor intron density relative to the corresponding minor intron density in human. Because the use of human as the outgroup for reconstructions of fungal and plant ancestors results in very small absolute numbers of minor introns, kingdom-specific outgroups were chosen instead: the estimates of ancestral densities in fungi are relative to *Rhizophagus irregularis*, and those for plants are relative to *Lupinus angustifolius*. Because multiple species combinations were used to estimate the minor intron density at each ancestral node, we report the average value over all *n* estimates for each node as


\begin{eqnarray*} \overline{\rho }_{minor}=\frac{1}{n} \sum _{i=1}^{n} \hat{\rho }_{minor_i}, \end{eqnarray*}

and the value relative to a reference species as the average ± the standard error of the mean (Figure 8).

To concretize this process with a specific example, in reconstructing minor intron density for the Chordata-Echinodermata ancestor we used (among others) alignments of orthologs from *Homo sapiens* (α), the acorn worm *Saccoglossus kowalevskii* (β) and the sea slug *Aplysia californica* (γ) as an outgroup. Within these alignments, we found 127 minor introns shared between *H. sapiens* and *S. kowalevskii* (*N*_αβ_), 116 between *H. sapiens* and *A. californica* (*N*_αγ_), 136 between *S. kowalevskii* and *A. californica* (*N*_βγ_) and 102 between all three species (*N*_αβγ_). Given these values, the number of ancestral minor introns in the aligned genes $\hat{N}_{\Omega _{minor}}$ is estimated as


\begin{eqnarray*} \hat{N}_{\Omega _{minor}} = \frac{{127 \cdot 116 \cdot 136}}{102^2} \approx 193. \end{eqnarray*}

For major introns, the same procedure yields


\begin{eqnarray*} \hat{N}_{\Omega _{major}} = \frac{{14220 \cdot 14800 \cdot 14954}}{13062^2} \approx 18446, \end{eqnarray*}

resulting in a final estimate—based on these alignments—of minor intron density in the Chordata-Echinodermata ancestor


\begin{eqnarray*} \hat{\rho }_{minor}=\frac{193}{193 + 18446} \cdot 100\% \approx 1.035\%. \end{eqnarray*}

Dividing this estimate by the minor intron density found in the aligned human genes ($\approx 0.828\%$) results in a relative density estimate of ≈1.25 (i.e. a roughly 25% enrichment) versus the minor intron density in human (Figure 8).

As has been pointed out in other contexts, ancestral state reconstructions may be confounded by several different factors (54–56). Many such concerns are minimized in our specific approach given that (a) the traits under consideration are not complex, but rather the simple binary presence/absence of discrete genetic elements, (b) calculations are restricted to introns present in well-aligning regions of orthologs (thereby avoiding issues with missing gene annotations in a given species, since alignments must include sequences from all species to be considered) and (c) the contribution of parallel intron gain, especially of minor introns, is likely to be very small (57–59). There are a number of other potential sources of bias in our analyses, however, which are worth addressing.

First, our ancestral intron density estimates are (to a large, though not complete, extent) dependent upon the accuracy of the phylogenetic relationships shown in Supplementary Figure S1. Ideally, we would have perfect confidence in all of the relationships underlying each node’s reconstruction, but such an undertaking is beyond both the scope of this paper and the expertise of its authors. While we have done our best to be assiduous in choosing nodes with well-resolved local phylogenies—which is one reason similar reconstructions have not been provided for a much larger number of nodes from less-confident phylogenetic contexts—it remains the case that our reconstructions are only fully informative with respect to the tree upon which they are based. That being said, unless the phylogeny for a given node is so incorrect as to have mistaken one of the ingroups for the outgroup (i.e., the chosen outgroup was not in fact an outgroup), the reconstruction should still represent the ancestor of the two ingroup species.

Second, we are relying on the correct identification of minor introns within each species to allow us to identify conserved/non-conserved minor introns in multi-species alignments. Although the field in general lacks a gold-standard set of verified minor introns upon which to evaluate classifier performance, the low empirical false-positive rate of intronIC (as determined by the number of minor introns found in species with compelling evidence for a lack of minor splicing) and the high degree of correspondence of its classifications with previously-published data suggests that our analyses are capturing the majority of the minor introns in each alignment.

There is also the possibility that many minor introns are unannotated in many genomes (and in fact, for certain annotation pipelines we know that this has historically been the case). This concern is mediated somewhat by the fact that, because we are only considering gene models that produce well-aligning protein sequences across multiple species, our alignments are unlikely to contain unannotated introns of either type. Unannotated minor introns, necessarily residing in unannotated genes would of course not be considered in our analyses, which would reduce the total number of orthologous genes compared and might raise concerns that the chosen samples may not reliably represent the complete data with sufficient confidence. We have done what we can to combat this by choosing species with annotations of high quality (as assessed by BUSCO completeness, for example), and by using multiple combinations of species to reconstruct each node. In reconstructions based upon large numbers of different alignments, the low standard errors of the estimates give us some confidence that missing data of this kind is unlikely to qualitatively affect our results.

### Differential gene expression

Single-end RNA-seq reads from previously-published cell-type-specific sequencing of *Rhizophagus irregularis* (60) (four biological replicates per cell type) were pseudoaligned to a decoy-aware version of the transcriptome using Salmon v1.6.0 (61) (with non-default arguments - -seqBias- -softclip). The Salmon output was then formatted with tximport v1.14.2 (62), and differential gene expression (DGE) analysis was performed using DESeq2 v1.26.0 (63) with the following arguments: test = ‘LRT’, useT = TRUE, minReplicatesForReplace = Inf, minmu = 1e-6, reduced = ∼ 1. For each pairwise combination of cell types, genes with significant DGE values (Wald *P* < 0.05) were retained for further analysis.

### *Rhizophgaus* z-score metric

Following the methodology used by Sandberg et al. to assign a proliferation index to cell types (64), z-scores were calculated per feature (whether for gene expression or intron retention/splicing efficiency) across all cell-type replicates (*n* = 20), and then summarized for each cell type by the mean value of the corresponding replicate z-scores (departing from the reference method in this aspect). Prior to conversion to z-scores, the raw gene expression data was normalized by running the output from tximport through the fpkm function in DESeq2. For group z-score comparisons (e.g., proliferation-index genes, minor introns versus major introns), the median of the top 50% of z-scores from each group was used. As the z-score calculation requires there to be variation across samples, certain genes/introns were necessarily omitted under this metric.

### Intron retention and splicing efficiency

For each RNA-seq sample, IRFinder-S v2.0 (65) was used to compute intron retention levels for all annotated introns. Introns with warnings of ‘LowSplicing’ and ‘LowCover’ were excluded from downstream analyses. Across replicates within each cell type, a weighted mean retention value was calculated for each intron, with weights derived by combining the average number of reads supporting the two intron-exon junctions and the total number of reads supporting the exon-exon junction.

Intron splicing efficiency was calculated as previously described (20). Briefly, RNA-seq reads were mapped to splice-junction sequence constructs using Bowtie v1.2.3 (66) (excluding multiply-mapping reads using the non-default argument -m 1). Introns with fewer than five reads supporting either the corresponding exon-exon junction or one of the intron-exon junctions (or both) were excluded. For each intron, the proportion of reads mapped to the intron-exon junction(s) versus the exon-exon junction was used to assign a splicing efficiency value for each sample (see reference for details). Within each cell type, the weighted mean of replicate splicing efficiency values for each intron was calculated in the same manner as for intron retention.

### Spliceosome-associated gene expression

Orthologs of human spliceosome components were found in *Rhizophagus irregularis* via a reciprocal-best-hit approach (https://github.com/glarue/reciprologs) using BLAST v2.9.0+ (67) with an E-value cutoff of 1 × 10^−10^. Four genes from each splicing system (major and minor) were identified in *Rhizophagus* by this approach, consisting of orthologs to human minor spliceosome genes ZMAT5 (U11/U12-20K), RNPC3 (U11/U12-65K), SNRNP35 (U11/U12-35K) and SNRNP25 (U11/U12-25K) and major spliceosome genes SF3A1 (SF3a120), SF3A3 (SF3a60), SNRNP70 (U1-70K) and SNRPA1 (U2 A′). Gene expression values generated by Salmon for each set of genes in each cell type were averaged across replicates, and pairwise comparisons between cell types were made for the same set of genes (e.g., minor spliceosome genes in IS versus MS). The significance of differences in expression between paired gene sets from different cell types was assessed using a Wilcoxon signed-rank test, with *P*-values corrected for multiple testing by the Benjamini–Hochberg method.

### Publicly-available minor intron database

Intron metadata for all species identified as containing minor introns in our analyses was collected, including summary statistics for all annotated genes (such as genic intron density and gene length), taxonomic classification information for each species based on the NCBI Taxonomy Database (37) and links to the source databases for the genome and annotation files. This data was then structured according to a chosen database schema in CSV format and converted to an SQLite database using sqlite-utils v3.32.1 (https://github.com/simonw/sqlite-utils). A virtual private server was established to host the SQLite database, and datasette v0.64.3 (https://github.com/simonw/datasette) was used to enable public access the database via an interactive web interface at https://www.introns.info.

A particular advantage of the approach we have implemented is the ability for users of the database to download selected subsets of the data in accessible formats. The results of a given query, for example, can be downloaded in a variety of plain text formats, and the entire SQLite database file (≈40 GB) can be downloaded directly from the web interface. A compressed version of the same database has been deposited in Dryad at https://doi.org/10.6071/M36Q39.

## Results

### Minor intron diversity in thousands of eukaryotic genomes

In order to better assess the landscape of minor intron diversity in eukaryotes, we used the intron classification program intronIC (19) to process ≈270 million intron sequences and catalog minor intron presence (or absence) in over 3000 publicly-available eukaryotic genomes, representing to our knowledge the largest and most diverse collection of minor intron data assembled to date (Figure 1, Supplementary Figure S1).

**Figure 2.**
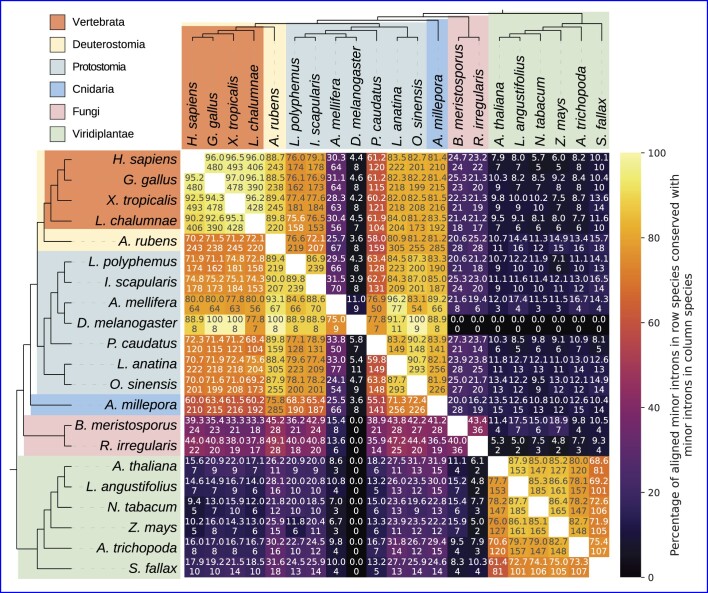
Pairwise minor intron conservation between various species. Bottom number is the number of minor introns conserved between the pair; top number is the number of conserved minor introns as a percentage of the minor introns present in the alignments for the associated species (the row species). For example, there are eight minor introns conserved between *D. melanogaster* and *L. polyphemus*, which is 88.9% of the *Drosophila* minor introns present in the alignment, but only 4.3% of the corresponding minor introns in *Limulus*. Full names of species are as follows: *Homo sapiens*, *Gallus gallus*, *Xenopus tropicalis*, *Latimeria chalumnae*, *Asterias rubens*, *Limulus polyphemus*, *Ixodes scapularis*, *Apis mellifera*, *Drosophila melanogaster*, *Priapulus caudatus*, *Lingula anatina*, *Octopus sinensis*, *Acropora millepora*, *Basidiobolus meristosporus*, *Rhizophagus irregularis*, *Arabidopsis thaliana*, *Lupinus angustifolius*, *Nicotiana tabacum*, *Zea mays*, *Amborella trichopoda*, *Sphagnum fallax*.

Of the 1844 genera represented in our data, 1172 (64%) have well-supported evidence of minor introns in at least one species (see Materials and methods for details; underlying plain text data available at https://doi.org/10.6084/m9.figshare.20483655), while the remaining 672 appear to lack minor introns in all available constituent species (Supplementary Figures S1 and S2). Consistent with previous studies (19,21,22,31,34,35,69–71), minor intron numbers and densities (fractions of introns in a given genome classified as minor type) vary dramatically across the eukaryotic tree; average values are highest in vertebrates and other animals, while variation between species appears to be lowest within land plants. Conservation of minor introns between different pairs of species is largely consistent with previously-published results (19,21,22,34) (Figure 2). The intriguing pattern of punctuated wholesale loss of minor introns is apparent within many larger clades in our data, along with a number of striking cases of minor intron enrichment in otherwise depauperate groups.

#### Minor intron enrichment

As shown in Figure 1 and Supplementary Figure S1, the highest known minor intron density is found within the Amoebozoa; our recently-reported data in the slime mold *Physarum polycephalum* (20) dwarfs all other known instances of local minor intron enrichment and appears to be an extremely rare example of significant minor intron gain. In the present study, we also find relatively high numbers of minor introns (compared to other amoebozoan species) in both the flagellar amoeba *Pelomyxa schiedti* (*n* = 90) and the variosean amoeba *Protostelium aurantium* (*n* = 265; incorrectly labeled as *Planoprotostelium fungivorum* in the NCBI database; see (72) for supporting evidence of its classification as *Pr. aurantium*). Although the numbers of minor introns in these species conserved as minor introns in other lineages (e.g. human) are very low, in all cases we find at least some degree of conservation. For example, in alignments between human and *P. aurantium* orthologs, 11% of human minor introns are conserved as minor introns in *P. aurantium*, comparable to proportions shared between human and many plant species (19); in alignments with *P. schiedti* the proportion of conserved human minor introns is closer to 2.5%, although this seems to largely be due to massive minor-to-major conversion of ancestral minor introns in *P. schiedti*, as 69% of the human minor introns in those alignments share positions with major introns in *P. schiedti*.

As first reported by Gentekaki *et al.* (73), the parasitic stramenopile microbe *Blastocystis* sp. subtype 1 contains hundreds of minor introns, although our pipeline identifies $\approx 45\%$ fewer (*n* = 253) than previously described. Interestingly, the *Blastocystis* minor introns we do identify are highly enriched for the AT-AC subtype (77% or 196/253, compared to $\approx 26\%$ of minor introns in human), and the classic minor intron bias away from phase 0 is inverted, with 49% (124/253) of the putative minor introns in phase 0. *Blastocystis* also has the shortest average minor intron length in the data we analyzed at just under 42 bp (median 39 bp; introns shorter than 30 bp were systematically excluded in all species).

Surprisingly, we observe unusually high minor intron densities in a number of fungal species, a kindgom which until now was not known to contain significant numbers of minor introns. In particular, the Glomeromycete species *Rhizophagus irregularis* has a minor intron density comparable to that of humans (0.272%, *n* = 205), and *Basidiobolus meristosporus*, in the Zoopagomycota, has one of the highest minor intron densities outside of the Amoebozoa (0.554%, *n* = 249) (Figure 1). Consistent with earlier reports (23,34), we do not find any convincing support for minor introns in either of the two largest fungal groups, Ascomycota and Basidiomycota, which seem to have lost most if not all of the required minor snRNAs in the vast majority of species.

Our analysis confirms the presence of a small number of minor introns in the oomycete genus *Phytopthora* as described previously by other groups (14,34); in addition, we find that members of the stramenopile water mould genus *Saprolegnia* contain dozens of minor introns each (Figure 1). While any species with a very low reported number of minor introns raises concerns about false positives, subsets of minor introns from each of these lineages have been found in conserved positions with minor introns in distantly-related species in our data, and the presence of minor snRNAs in each of the aforementioned genomes provides further evidence for the existence of bona fide minor introns in these species (Figure 1). Interestingly given its sister placement to the broadly minor-intron-poor nematode clade, the cactus worm *Priapulus caudatus* appears to be quite minor-intron rich (*n* = 330, 0.316%), with substantial minor intron conservation to other metazoan lineages (Figure 2).

Within the protostomes, one of the two sister clades of bilateria, there are cases of relative minor intron enrichment in both arachnids (Arachnida) and molluscs (Mollusca) (Figure 1, Supplementary Figure S1), as well as in the brachiopod species *Lingula anatina* and the horseshoe crab *Limulus polyphemus*. Ixodida, including *Ixodes scapularis*, *Dermacentor silvarum* and *Rhipicephalus*, has a much higher average minor intron density than other groups within Acari, which includes both mites and ticks and is generally very minor-intron poor.

On the other side of the bilaterian tree, minor intron densities in deuterostomes are far more homogeneous. Vertebrates have consistently high minor intron densities ($\approx 0.3\%$), and the remaining deeply-diverging clades within deuterostomes—with the exception of tunicates, which appear to have lost a significant fraction of their ancestral minor intron complement—have minor intron densities comparable to vertebrates (e.g. the starfish *Asterias rubens*).

In their seminal paper examining spliceosomal snRNAs in diverse eukaryotic lineages, Dávila López *et al.* (23) described a number of clades missing most/all of the usual minor snRNAs. Based upon our larger dataset, it now seems clear that at least some of these groups do in fact have both minor introns and many if not all of the canonical minor snRNAs. These include the *Acropora* genus of coral, which has an average minor intron density higher than that of most vertebrates; within fungi the Chytridiomycete species *Spizellomyces punctatus* as well as a number of Neocallimastigomycetes including *Piromyces finnis* and *Neocallimastix californiae*; the genus of blood flukes *Schistosoma*; and all of the species of Streptophyta included in the earlier analysis (see Figure 1 in (23)). Notably, we also find minor introns (verified by comparative genomic methods) in the green algal species *Chara braunii* (*n* = 166) and *Klebsormidium nitens* (*n* = 110), representatives of a group which until now was thought to lack minor splicing entirely (14,23,34), as well as in the Glaucophyte alga *Cyanophora paradoxa* (*n* = 77) (which may have transformed minor splicing machinery, as we find significant hits to only the U11 snRNA in that species) (Figure 1).

#### Minor intron depletion

Punctuated and dramatic loss of minor introns is a hallmark feature of the minor splicing landscape, and it remains an outstanding question why certain lineages undergo either partial or complete loss of their ancestral minor introns while others do not (71). Previous work has delineated many groups that appear to lack either minor introns, minor splicing components or both (14,23,34), but the diversity and scope of more recently-available data motivated us to revisit this topic. Within the aggregate data presented in Figure 1 and Supplementary Figure S1, there are a number of cases of severe or complete minor intron loss that we highlight here. First, the amoebozoan *Acanthamoeba castellanii* has been found to contain both minor splicing apparatus as well as a limited number of introns with minor-like sequences (14). While it remains likely that this species contains a small number of minor introns based upon previous evidence, none of the twelve *Acanthamoeba* introns our pipeline classified as minor were found to be conserved in either human or the more closely-related amoeobozoan *Protostelium aurantium*. We do, however, find a single shared minor intron position between *Acanthamoeba* and human when we disregard local alignment quality and simply consider all introns in identical positions within aligned regions, which amounts to 20% of *Acanthamoeba* minor introns in such alignments.

Notable examples in our data of clades with extreme but incomplete loss (of which Diptera is a classic case in animals) include the bdelloid rotifers, the springtail (Collembola) subclass of hexapods as well as the Acari (ticks and mites). The latter, in addition to its extreme reduction of minor introns generally also appears to contain a number of cases of complete (by comparative genomic analysis) loss in the parasitic mite *Tropilaelaps mercedesae* and the earth mite *Halotydeus destructor*. Furthermore, we find no evidence at all for minor introns in the following taxa, a number of which have not to our knowledge been reported before (those with associated citations corroborate earlier studies): tardigrades (e.g. *Hypsibius exemplaris*), Discoba (e.g. *Trypanosoma*, *Leishmania*) (23), Orchrophyta (stramenopiles), Alveolata (protists) (23,34). We also report two other novel cases of apparent complete minor intron loss outside of Acari. First, in the Dipteran clade Chironomidae, there is little evidence of minor intron presence in *Clunio marinus*, *Polypedilum vanderplanki* and *Belgica antarctica*, all of which also seem to be missing between half and three-quarters of their minor snRNAs. Second, in our data the copepod crustaceans *Tigriopus californicus* and *Eurytemora affinis* each lack conserved minor introns and 75% of the canonical minor snRNA set (Figure 1, Supplementary Figure S1).

### Minor introns have lower average conservation than major introns

A persistent result in the minor intron literature is that minor introns are more highly conserved than major introns (specifically, between animals and plants and even more specifically, between human and *Arabidopsis thaliana*) (74), although this assertion has been contradicted by at least one more recent analysis (19). The claim that minor intron conservation exceeds major intron conservation rests largely upon the numbers of introns of both types found in identical positions within 133 alignments of orthologous human-*Arabidopsis* sequences, as reported in Table 1 of Basu *et al.* (74). For major introns, the authors report 115 conserved as major in aligned ortholog pairs, and 1391 as either not present in one of the paired orthologs or present as a minor intron; for minor introns, they find 20 conserved and 135 missing/converted. For each intron type, taking the number conserved and dividing by the total number of introns of that type present in the alignments results in conservation percentages of 7.6% ($\frac{115}{115 + 1391}$) for major introns and 12.9% ($\frac{20}{20 + 135}$) for minor introns (although these summary statistics themselves are not presented explicitly in the text). This data, then, would appear to support the conclusion that minor introns are more highly conserved between human and *Arabidopsis* than are major introns. To the extent that we correctly understand the previous approach, however, we believe there may be a complication with this analysis.

**Table 1.** Comparison of major and minor intron conservation between human and *Arabidopsis thaliana*

Major			Minor			
*N*_*cons*_	*N*_*var*_	Conservation (%)	*N*_*cons*_	*N*_*var*_	Conservation (%)	*p*_*Fisher*_
2052	14 162	12.7	7	120	5.5	0.015

N_cons_ indicates the number of introns of each type conserved as the same type in both human and *Arabidopsis*. *N*_var_ indicates the total number of introns (of both species) present in the alignments where the corresponding position in the opposing sequence either does not contain an intron, or contains an intron of the other type.

**Figure 3.**
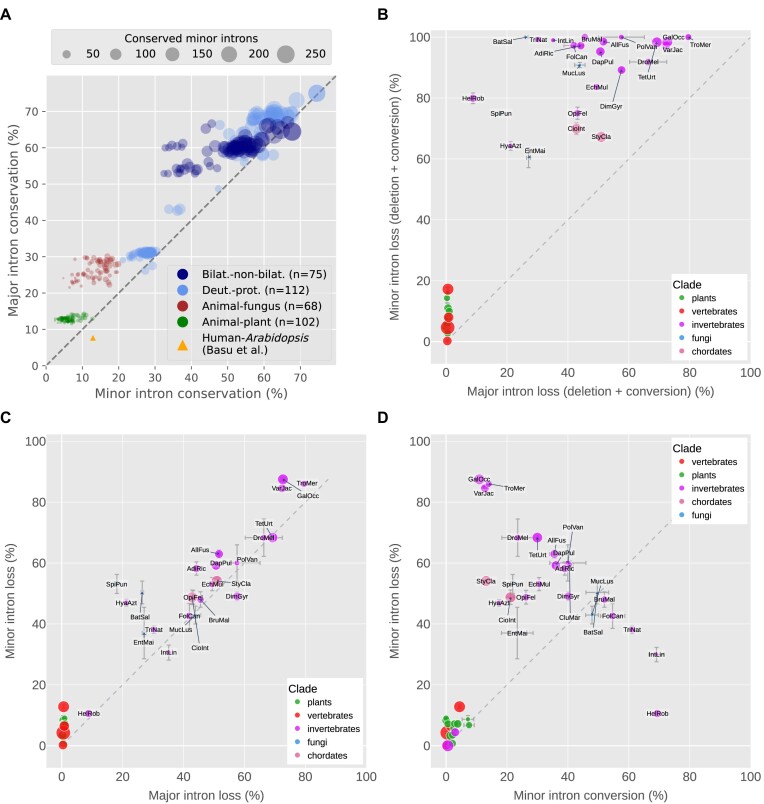
Conservation and loss of minor and major introns. (**A**) Comparison of major (y-axis) versus minor (x-axis) intron conservation across hundreds of pairs of species. Bilat.-non-bilat.: bilaterian versus non-bilaterian (animal); Deut.-prot.: deuterostome versus protostome. The yellow triangle indicates levels of conservation of major and minor introns between *Homo sapiens* and *Arabidopsis thaliana* as reported by Basu *et al.* (74). Size of markers indicates number of minor introns conserved between each pair. (**B**) Minor versus major intron loss, where ‘loss’ includes both sequence deletion and conversion to an intron of the other type. Bars indicate standard error of the mean for averaged values. Marker size represents relative minor intron density. (**C**) Minor versus major intron loss, where ‘loss’ represents actual deletion of the intron sequence. (**D**) Minor intron loss versus conversion, where ‘loss’ represents actual deletion of the intron sequence. Species abbreviations for are as follow: AdiRic: *Adineta ricciae*, AllFus: *Allacma fusca*, BatSal: *Batrachochytrium salamandrivorans*, BruMal: *Brugia malayi*, CioInt: *Ciona intestinalis*, CluMar: *Clunio marinus*, DapPul: *Daphnia pulicaria*, DimGyr: *Dimorphilus gyrociliatus*, DroMel: *Drosophila melanogaster*, EchMul: *Echinococcus multilocularis*, EntMai: *Entomophaga maimaiga*, FolCan: *Folsomia candida*, GalOcc: *Galendromus occidentalis*, HelRob: *Helobdella robusta*, HyaAzt: *Hyalella azteca*, IntLin: *Intoshia linei*, MucLus: *Mucor lusitanicus*, OpiFel: *Opisthorchis felineus*, PolVan: *Polypedilum vanderplanki*, SpiPun: *Spizellomyces punctatus*, StyCla: *Styela clava*, TetUrt: *Tetranychus urticae*, TriNat: *Trichinella nativa*, TroMer: *Tropilaelaps mercedesae*, VarJac: *Varroa jacobsoni*.

Examining the orthologous alignments the authors provide in their supplementary data, it is evident that many of the same *Arabidopsis* sequences are present in multiple pairs of orthologs, which suggests that a standard reciprocal-best-hit criteria for ortholog identification was not employed and that certain introns will be counted multiple times within the overall set of alignments. As many minor introns occur in larger paralogous gene families, this methodology could lead to artificial inflation of the calculated minor intron conservation, especially given the small absolute number of minor introns present. To attempt to more thoroughly address the question of minor versus major intron conservation, we identified orthologs in many different pairs of species across a range of evolutionary distances (see Materials and methods), and calculated intron conservation using the same metric as the previous work. Across more than 100 such comparisons between animals and plants (and more than 60 between animals and fungi), we find no cases where minor intron conservation exceeds major intron conservation (Figure 3A).

Furthermore, in alignments of more closely-related species we observe only a handful of cases where minor intron conservation marginally exceeds major intron conservation ( e.g., $\approx 3\%$ greater between the starfish *Asterias rubens* and the stony coral *Orbicella faveolata*, Figure 3A,). Lastly, in the specific case of human-*Arabidopsis* considered by Basu et al., our more recent data show minor intron conservation to be less than half that of major intron conservation (Table 1). Thus, in the final analysis we find no compelling support for the idea that minor introns are in general more conserved than major introns and in fact, the opposite seems to be true in the vast majority of cases.

### Minor intron loss versus conversion

When an ancestral minor intron ceases to be a minor intron, it is thought to happen primarily in one of two ways: the entire intron sequence could be lost via, for example, reverse transcriptase-mediated reinsertion of spliced mRNA (21,71,75,76), or the intron could undergo sequence changes sufficient to allow it to be recognized instead by the major spliceosome (31,77–79). From first-principles arguments based on the greater information content of the minor intron motifs (31,33,77) along with limited empirical analyses (21), it is assumed that intron conversion proceeds almost exclusively unidirectionally from minor to major. Previous work has also shown that the paradigm of full intron loss (sequence deletion) appears to dominate over conversion in minor introns (21), a pattern we were interested to explore further in our expanded dataset.

First, we assembled a manually-curated sample of species with significant/complete minor intron loss, along with a number of species with much higher minor intron conservation for comparison. For each selected species, we chose an additional species to compare against as well as a species to serve as an outgroup, and then identified orthologs between all members of the set to allow us to identify ancestral introns (see Materials and methods for details) and estimate fractions of each intron type retained from the ancestral complement. Considering loss to include both sequence deletion as well as type conversion (which we assume to be unidirectional from minor to major, as discussed above), we found minor intron loss to be more pronounced than major intron loss in the species we examined (Figure 3B; shown more generally in Figure 3A).

We can also decompose the phenomena contributing to the higher degree of loss in minor introns and ask whether the rate of sequence deletion specifically, for example, differs between the two intron types. Somewhat surprisingly, we find that this form of loss is very similar between the two types of introns in species which have lost significant fractions of their minor introns (Figure 3C). Because species were chosen based upon putative loss of minor introns and the sample size is low, it is difficult to interpret the apparent bias toward minor intron deletion in the vertebrates and plants included in Figure 3C. Nevertheless, for the other species this data suggests that in instances of pronounced minor intron upheaval there is not a particular selective pressure to remove minor intron sequences themselves—at least not any more than there is pressure to remove intron sequences generally.

In addition, we can look at the other form of minor intron ‘loss’, conversion from minor to major intron type. Here, we find that in many instances loss via deletion does indeed outstrip conversion (as reported by (21)), sometimes dramatically so, but there are interesting exceptions. The leech *Helobdella robusta* (HelRob), for example, which seems to have retained a large fraction of its ancestral major introns (80), has lost $\approx 80\%$ of its minor introns primarily through conversion to major type (Figure 3D). By contrast, the annelid worm *Dimorphilus gyrociliatus* (DimGyr), found in a clade (Polychaeta) sister to *Helobdella*, has undergone a seemingly independent loss of minor introns of similar proportion to *Helobdella* under a very different modality, with loss (deletion) outweighing conversion (Figure 3D). It is unclear what forces are responsible for the relative contributions of each mechanism; in *Helobdella*, the major intron sequences are slightly more degenerate at the 5′SS end than in e.g., human, which might lower the barrier to entry for would-be minor-to-major converts. This is speculation, however, and more work is needed to better characterize these dynamics. It should be noted that under the current analysis we cannot differentiate between losses, and conversions followed by subsequent loss; our conversion estimates should therefore be taken as conservative.

### Positional biases of major and minor introns

It has been known for many years that introns often exhibit a 5′ bias in their positions within transcripts (81–83). This can be explained in large part due to biased intron loss: because a primary mechanism of intron loss is thought to occur via the reverse-transcriptase mediated (and 3′-biased) insertion of spliced mRNA (84–86), over time such a process would tend to result in higher concentrations of introns closer to the 5′ ends of transcripts.

Less attention has been paid to the positional biases of minor introns specifically, although at least one study (74) found that minor introns appear to be especially over-represented in the 5′ portions of transcripts in both human and *Arabidopsis thaliana*. We were curious to see whether the same patterns were present in our own data and whether they generalized beyond the two species so far examined.

**Figure 4.**
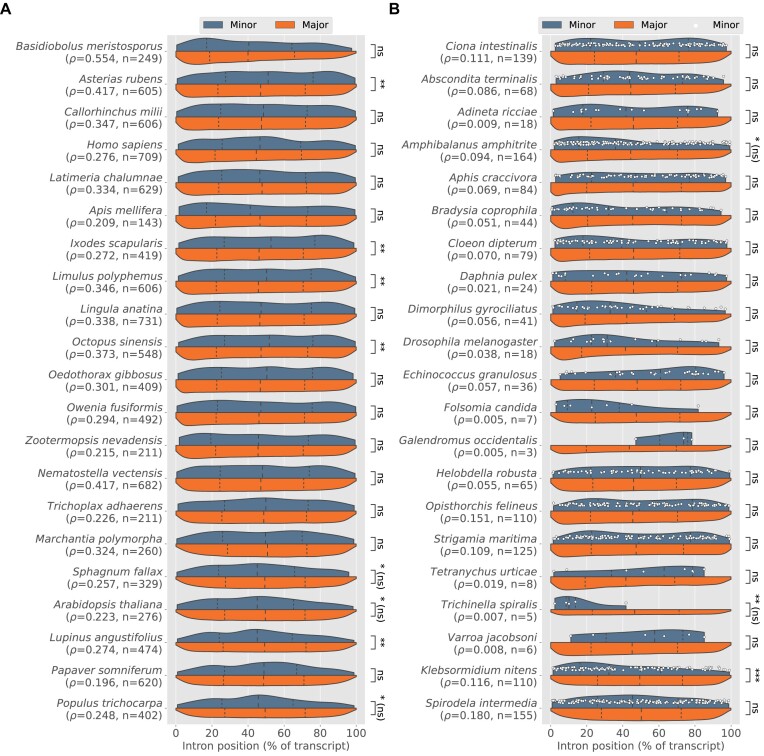
Intron position distributions for major (red) and minor (yellow) introns in selected species. (**A**) Species enriched in minor introns. (**B**) Species with significant inferred minor intron loss; white dots represent individual minor introns. For both plots: Dashed lines represent the first, second and third quartiles of each distribution; ρ indicates minor intron density; *n* is number of minor introns; statistically significant differences between minor and major introns are indicated with asterisks (two-tailed Mann–Whitney *U* test; *P ≤ 0.05; **P ≤ 0.001; ***P ≤ 0.0001; "ns" not significant; asterisks followed by "(ns)" indicate statistical significance assignments that did not survive correction for multiple testing under Benjamini–Hochberg). Note that in some cases of significant difference between the two intron types, e.g. within animals, it is the *major* introns with greater 5′ bias.

We selected two sets of species to highlight—for the first, we chose lineages with substantial numbers of minor introns from a variety of groups; for the second, we picked species with significant amounts of inferred minor intron loss to investigate whether any 5′ bias might be more extreme in the remaining minor introns. In our analysis, we confirm the 5′ bias as previously described (74) in *Arabidopsis thaliana* (Figure 4A), although we do not find the same difference as described in the earlier study between major and minor intron positions in human.

Overall, our results point to a less-clear picture than previous work might suggest. While we do find a number of cases in animals where minor introns are more 5′-biased than major introns (Figure 4B, *Amphibalanus amphitrite* and *Trichinella spiralis*), the pattern is not broadly significant and is occasionally reversed, albeit only significantly so in animal species with less-dramatic minor intron loss (e.g. *Ixodes scapularis*, *Asterias rubens*, Figure 4A). Within plants, however, a clearer pattern is apparent, with a higher fraction of plants species in both groups displaying a strong 5′ bias in their minor introns. To determine how widespread this pattern of greater relative 5′ bias in minor introns is, we searched our entire dataset for species with a) significant differences in minor intron occurrence between the 5′ and 3′ halves of trancripts (assessed as in (74) with a two-tailed exact binomial test, where presence in the 5′ half of a transcript was considered a success), (b) significant differences between major and minor intron positions as determined by a two-tailed Mann–Whitney *U* test and c) median minor intron positions more 5′ biased than median major intron positions (as the first two tests do not provide information about which intron type is biased in which direction). Among species meeting these criteria, plants were significantly over-represented (Table 2, *P* = 8.9 × 10^−68^ by a Fisher’s exact test). The *P*-values for the Mann–Whitney *U* tests supporting the data in Table 2 have not been corrected for multiple testing, as our conclusions do not depend on the significance of any individual result. The same qualitative pattern, however, is also found after multiple testing correction (in which case only 11 species, all in Streptophyta, are found with significant 5′ minor intron bias; Benjamini–Hochberg method; Fisher’s exact *P* = 6.81 × 10^−09^).

**Table 2.** Proportions ($\%$) of species in various groups with statistically-significant 5′ bias of minor intron positions within transcripts ($N_{5^{\prime }{} MIB}$)

Clade	*N*_*total*_	$N_{5^{\prime }{} MIB}$	%
Streptophyta	290	112	38.6
Fungi	63	2	3.2
Metazoa	1204	21	1.7
Stramenopiles	16	0	0.0
Evosea	4	0	0.0
Discosea	1	0	0.0

It is possible that this pattern, taken together with the higher degree of stability of minor intron densities in plants, reflects an ancient loss of minor introns in the plant ancestor, the signature of which is now shared broadly among extant species. It may also suggest a unique and/or more consistent paradigm of minor intron loss in plants, distinct from the relatively haphazard process seemingly at work within other parts of the eukaryotic tree where minor intron losses have occurred both more recently and more frequently.

### Phase biases of minor introns

**Figure 5.**
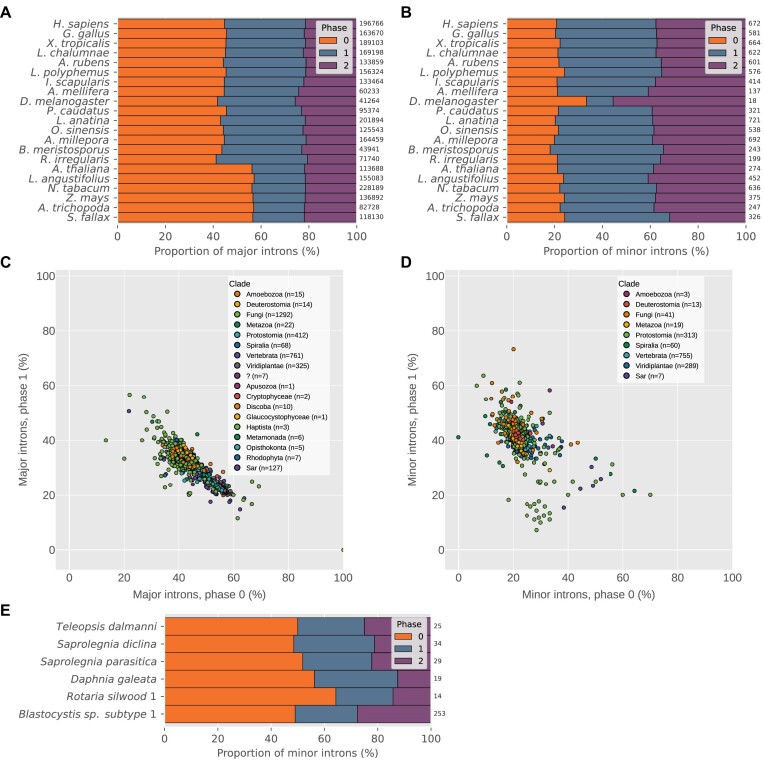
Minor and major intron phase biases. (**A**,**B**) Phase distributions of major and minor introns, respectively, in various species. Numbers at the ends of bars represent the total number of constituent introns. (**C**) Proportions of phase 1 (y-axis) versus phase 0 (x-axis) major introns. Correlation of phase 0 to phase 1 ρ_*s*_ = −0.81, *p* ≪ 0.0001. (**D**) Proportions of phase 1 (y-axis) versus phase 0 (x-axis) minor introns in species with at least 10 high-confidence minor introns. Correlation of phase 0 to phase 1 ρ_*s*_ = −0.48, *p* ≪ 0.0001. (**E**) Unusually high proportions of phase 0 minor introns in certain species (graphical elements as in (A) and (B)). Proportions of phase 0 minor introns for all species are significantly different from expected values derived from the proportion of phase 0 minor introns in human (phase 0 versus sum of other phases, Boschloo’s exact test *P* < 0.05). Species in (C) and (D) with fewer than 10 identified introns of the corresponding type were excluded, as were species in (D) with uncertain/borderline minor intron presence (see Curation of minor intron data/edge cases).

Spliceosomal introns can occur at one of three positions relative to protein-coding sequence: between codons (phase 0), after the first nucleotide of a codon (phase 1) or after the second (phase 2). In most species, major introns display a bias toward phase 0 (87,88) (Figure 5A), while minor introns are biased away from phase 0 (19,31) (Figure 5B).

It remains an unsettled issue why minor introns are biased in this way—one theory proposed by Moyer *et al.* (19) suggests that such a bias could arise from preferential conversion of phase 0 minor introns to major-type, which over time would lead to the observed pattern. Here, we made use of the size of our dataset to better characterize the diversity of intron phase patterns within each intron type. As shown in Figure 5C, the phase distributions of major introns are fairly tightly grouped; in our aggregate data, phase 0 makes up 44.3%, phase 1 31.2% and phase 2 24.5% (though we note here for posterity the most striking case of major intron phase bias we have observed in the yeast species *Candida maltosa*, which lacks minor introns, where all ≈1000 annotated major introns appear to be phase 0: Figure 5C, bottom-right corner). In addition, the proportions of phase 0 and phase 1 major introns are quite highly correlated (see caption of Figure 5C). Minor introns, on the other hand, are less consistent in their phase distributions and have a lower phase 0 to phase 1 correlation, although the majority cluster relatively tightly around the average value of 22% for phase 0 (Figure 5D).

It is intriguing that a small number of species appear to have much higher fractions of phase 0 minor introns (Figures 5D and E). What’s more, these species (with the notable exception of *Blastocystis* sp. subtype 1, addressed below) all have very low absolute numbers of minor introns (Figure 5E). While these data are not necessarily incompatible with the conversion paradigm mentioned above (which might predict minor introns in species with pronounced loss to show especially strong bias away from phase 0), they at least invite further investigation into the forces underlying the phase biases of minor introns generally.

The species *Blastocystis* sp. subtype 1 is similar to the other unusual cases mentioned in its atypically weak minor intron bias away from phase 0, but is remarkable for the number of minor introns involved (*n* = 253). Interestingly, its minor intron phase distribution is almost identical to the phase distribution of its major introns (not shown). While this raises the possibility that the minor introns in *Blastocystis* sp. subtype 1 are false-positives, the fact that we find (a) all four minor snRNAs in the genome, (b) a (small but non-zero) number of its minor introns conserved in *Lingula anatina* (not shown) and (c) putative minor introns in a closely-related species (*Blastocystis hominis*) provides evidence for their identity as real minor introns. Assuming they are bona fide minor introns, another possible explanation for their relative phase 0 enrichment could be that they have been more recently gained, and (under the conversion hypothesis) have not yet had time to develop the phase bias present in older minor intron sets. More thorough comparative genomics work within the clade after additional species become available would help to clarify the evolutionary picture.

### Non-canonical minor intron splice boundaries

The vast majority (> 98.5%) of major introns in most eukaryotic genomes begin with the dinucleotide pair GT, and end with the pair AG (16,19,35,89), with an additional smaller contingent of GC-AG introns present in many genomes. When minor introns were first discovered, they were initially characterized largely by their distinct AT-AC termini (10,11). However, it was subsequently demonstrated that the majority of minor introns in most species in fact share the same terminal boundaries as major introns (31,77), although the AT-AC subtype may constitute a more significant fraction of minor introns in certain species (19,22,34,71,80). Over time, additional non-canonical (i.e. not GT-AG, GC-AG or AT-AC) subtypes of minor introns have been identified in various organisms (19,21,22,35,90), but these analyses have been limited to species with available minor intron annotations which until now was a relatively small set.

Because non-canonical introns do not (by definition) look like normal introns, it can be difficult to differentiate between biological insights and annotation errors when examining eukaryotic splice site diversity at scale. For example, a recent report on non-canonical introns in diverse species described significant enrichment of CT-AC introns in fungi (15). However, and as addressed briefly in the paper itself, CT-AC boundaries are the exact reverse-complement of the canonical GT-AG boundaries, and other sequence motifs reported in the data similarly match expectations for canonical introns annotated on the wrong strand. To combat issues of this sort, we first performed multiple within-kingdom protein-level alignments of various animal and plant species with high relative levels of annotated non-canonical minor introns. Conserved introns were then clustered across many different alignments to form conserved intron sets, which were filtered into type-specific (major, minor) sets by including only introns from sets where at least two introns of the same type were found (see Materials and methods for details). These sets of introns are much less likely to contain spurious intron sequences, although they also may not fully represent more recent or lineage-specific splice site changes and do not include introns from every species with non-canonical introns in our data.

**Figure 6.**
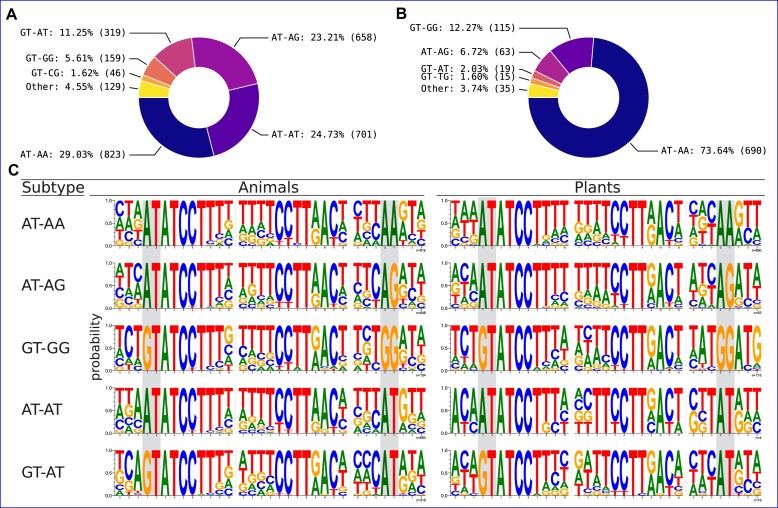
Non-canonical minor intron motifs in animals and plants. (**A**, **B**) Non-canonical intron termini found in conserved minor introns in animals and plants, respectively. Introns with non-canonical termini comprise $\approx 3.8\%$ of the total set of orthologous minor introns in animals and $\approx 4\%$ in plants (see Supplementary Table S1 for complete data including canonical minor introns). (**C**) Sequence logos of the 5′SS, BPS and 3′SS regions of selected non-canonical minor introns in animals and plants. The terminal dinucleotide pairs for each intron subtype are highlighted in gray.

Our results in animals (Figure 6A) and plants (Figure 6B) are largely consistent with previous data on non-canonical minor introns (21,35,90), with only small differences in the rank-order within each set. The assortment of non-canonical minor intron termini in plants is both less diverse and more lopsided than the animal set. For example, while the most common non-canonical termini is AT-AA in both kingdoms, almost 75% of all non-canonical minor introns we identify in plants are of this subtype, versus less than half of that proportion in animals. Conversely, the second most common non-canonical termini in animals, AT-AT, is almost entirely absent in plants.

As can be seen in Figure 6 and Supplementary Table S1, the majority of non-canonical termini differ by a single nucleotide from one of the canonical dinucleotide pairs. Additionally, there are small differences between the consensus sequences outside of the terminal dinucleotides across different subtypes of minor introns, and also within the same subtype between animals and plants (Figure 6C).

### Minor intron-containing genes are longer and more intron-rich than other genes

Across genomes in the eukaryotic tree, the number of introns contained in an average gene varies widely (85). For example, some vertebrate genes have dozens or even hundreds of introns (e.g. 363 introns in the human gene titin), whereas most genes in the yeast *Saccharomyces cerevisiae* lack introns entirely. Given the fact that minor introns appear to be arranged non-randomly within genomes (19,22,35,36) a question that arises is to what extent and in what ways are minor intron-containing genes (MIGs) different than those without minor introns? As far as we are aware, while various aspects of this question have been addressed by different groups (31,69,74), relatively little attention has been paid to possible differences in a number of basic gene attributes, namely gene length and number of introns per unit coding sequence or ‘genic intron density’ (a coinage we will use here to distinguish from our more frequent usage in this paper of ‘intron density’ to describe some number of introns in terms of their relative share of the total introns in the genome).

**Figure 7.**
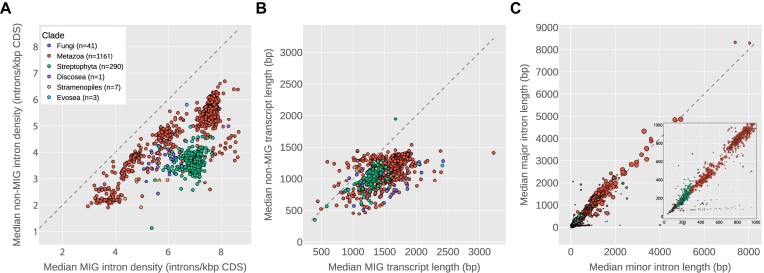
Features of MIGs and minor introns. (**A** and **B**) Median genic intron density (introns/kbp coding sequence) and gene length (sum of coding sequence), respectively, for major-intron-only genes (y-axis) versus minor intron-containing genes (x-axis). (**C**) Median major intron length (y-axis) versus median minor intron length (x-axis) for all species with high-confidence minor introns. Size of markers indicates number of minor introns in the genome. Inset: subset of the data with length ≤ 1000 bp. In all plots, species not confidently identified as containing at least ten minor introns were excluded. All three plots share the legend from (A).

Strikingly, within species containing minor introns, when we compare the genic intron density of MIGs to all other genes, we find that MIGs are universally more intron-dense on average than non-MIGs (Figure 7A). Furthermore, it appears that average MIG lengths (excluding intron sequences) are longer than other genes in the vast majority of species (Figure 7b). While there are a number of cases where the median non-MIG gene length exceeds the median MIG gene length, none of those differences are statistically significant (Mann–Whitney *U* test, *P* > 0.05).

The fact that minor introns are over-represented in older genes paired with the observation that older genes may skew longer and more intron-dense than younger genes (91,92) raises the possibility that the differences we report between MIGs and non-MIGs are merely a reflection of this differential age bias. We tested this hypothesis by constructing and examining sets of age-stratified genes in human, *Arabidopsis* and the fungus *Basidiobolus meristosporus* (see Materials and methods) and found that the pattern described above holds in all three genomes even when comparisons are limited to sets of genes within the same age categories (Supplementary Figure S3). Thus, it appears unlikely that biases in gene age alone could be responsible for the observed patterns. Although an in-depth analysis of this qualitative finding is beyond the scope of the current paper, it represents an underexplored distinction between the two intron types.

### Comparison of minor and major intron lengths

While a number of studies have compared the length distributions of different intron types in a limited assortment of genomes (19,36,93), without a large set of minor intron-containing species to compare within it has been difficult to gauge the extent to which minor intron lengths might differ from major intron lengths in general. With the comprehensive minor intron data we have collected, we were able to ask a very basic question: what is the typical relationship between average major and minor intron lengths? At a high level, the answer appears to be that major and minor intron lengths are roughly linearly correlated (Figure 7C)—species with longer average major intron length tend to also have longer average minor intron length (Spearman’s ρ = 0.903 for median values, *p* ≪ 0.0001). One interesting aspect of the data in Figure 7C is shown more clearly in the inset plot (which is the subset of the data in the main plot with length ≤ 1000 bp): certain species with significant minor intron loss (small markers) have relatively large differences between average minor and major intron lengths (major intron lengths ≲ 100 bp, minor intron lengths roughly 200–1000 bp). It should be noted that the set of species in that region is enriched for *Drosophila* (a genus taxonomically over-represented in the sequence databases), but includes many additional insect species as well.

Although it is not clear what immediate conclusions can be drawn from this data, some additional questions are raised: Were shorter minor introns especially selected against in these lineages, such that the remaining minor introns are disproportionately long? What is driving variation within, for example, *Drosophila* such that in some species the difference between minor and major is relatively modest (*Drosophila busckii*, major = 65 bp and minor = 189 bp) and in others, it’s much more stark (*Drosophila biarmipes*, major = 77 bp and minor = 677 bp)? It should be noted as well that for *Drosophila* specifically, almost all of the minor introns are conserved within the genus, so the previous example is made more compelling because 100% of the *D. busckii* minor introns are shared with *D. biarmipes*, yet are far longer in the latter than the former. It did occur to us to check whether minor introns in these outlier species happen to be (for whatever reason) in genes with longer-than-average intron size, and although we have not done so systematically an examination of a number of more extreme cases found the same pattern recapitulated between minor and major introns of the same genes. For example, in the black soldier fly *Hermetia illucens*, the median minor intron length is 4019 bp while the median major intron length is only 105 bp. Comparing minor to major within only the minor intron-containing genes changes things, but not qualitatively—the median major intron length increases to 399.5 bp, but the difference between minor and major is still significant (*P* = 0.0025 by a one-tailed Mann–Whitney *U* test under the alternative hypothesis that minor intron lengths are longer).

### Reconstruction of ancestral minor intron densities

**Figure 8.**
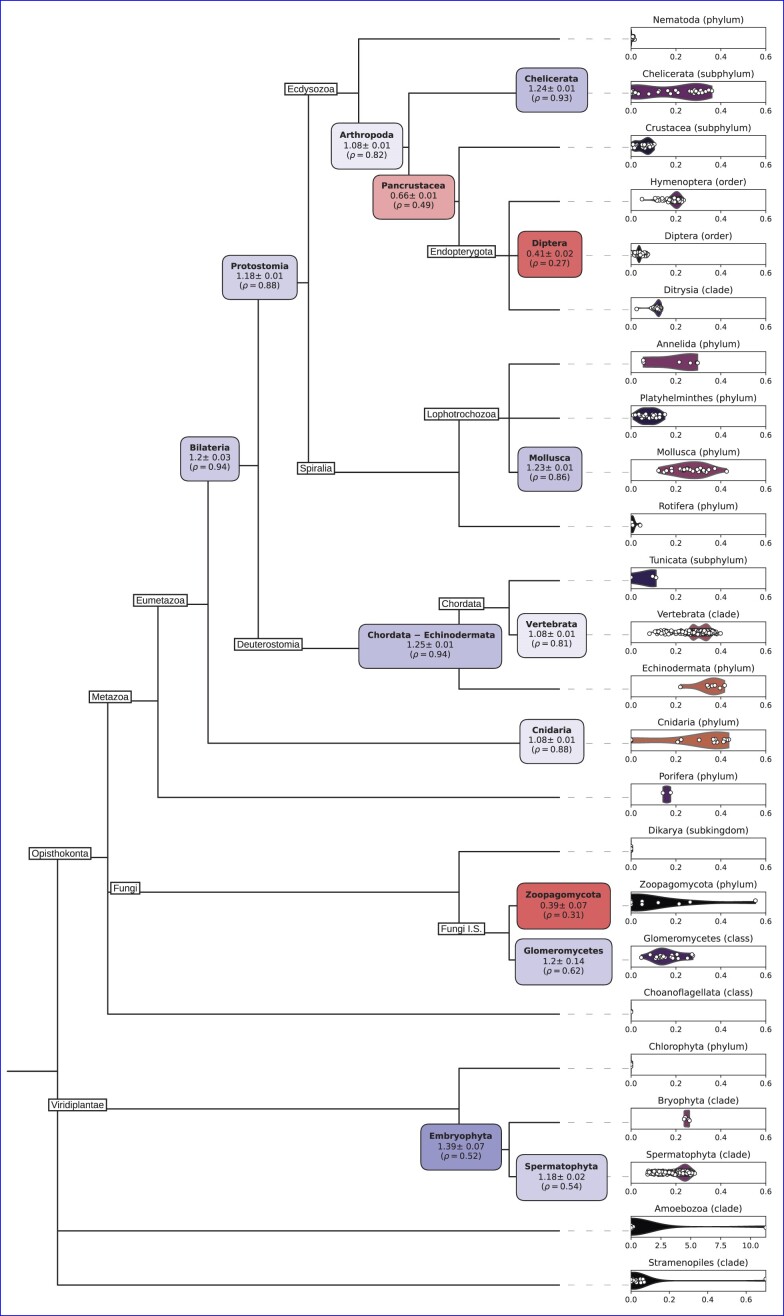
Minor intron density distributions in selected clades, and ancestral reconstructions of minor intron densities at selected nodes. Ancestral density node label color indicates enrichment (blue) or reduction (red) relative to the reference species in the alignments; the first number underneath each node label is the average estimated minor intron density at that node as a fraction of the reference species’ minor intron density; ρ indicates the node’s average estimated ancestral minor intron density. For animals, the reference species is *Homo sapiens*; for plants, *Lupinus angustifolius*; for fungi, *Rhizophagus irregularis*. Terminal violin plots show the distribution of minor intron densities (percent of all introns classified as minor) in extant lineages of the labeled taxonomic group.

In an attempt to quantify some of the evolutionary dynamics leading to the variegated pattern of minor intron densities found in extant lineages, we sought to estimate minor intron densities for certain ancestral nodes throughout the eukaryotic tree (see Materials and methods). For each selected node, we identified pairs of species for which the node is the most recent common ancestor and, in combination with an outgroup species, performed three-way protein-level alignments to allow us to define intron states for each species within the aligned sequences. Then, using the procedure described in (53), we calculated the number of minor and major introns estimated to have been present in the aligned regions in the ancestral genome (see Materials and methods), and repeated this process using many different combinations of species for each node to derive average values across all such comparisons. Because the absolute number of introns present in the aligned regions in the ancestor is not a particularly easy value to interpret, for reconstructions within a given kingdom we normalized the ancestral density of each intron type by a chosen reference species from that kingdom present in every alignment (see Materials and methods for details). The reference species for animals, fungi and plants were *Homo sapiens* (minor intron density 0.276%), *Rhizophagus irregularis* (minor intron density 0.272%) and *Lupinus angustifolius* (minor intron density 0.273%), respectively. Figure 8 shows distributions of minor intron densities in constituent species from each terminal clade (violin plots), as well as estimates of ancestral minor intron densities at various nodes (colored boxes) as fractions of the density of minor introns in the aligned regions of the reference species (i.e., ancestral densities > 1 indicate minor intron enrichment relative to the reference species, and ancestral densities < 1 indicate reduction).

As shown in Figure 8, ancestral minor intron densities were in large part modestly higher than the minor intron densities of the relatively minor-intron-rich reference species, with the exception of a number of episodes of pronounced loss in the ancestors of Diptera, Pancrustacea and Zoopagomycota. The apparent enrichment of minor introns in the ancestor of Chelicerata is interesting, as it suggests there may have been some amount of minor intron gain along that branch since the arthropod ancestor. This result needs to be qualified, however, by noting that we were constrained by lack of available data to using only *Limulus polyphemus* for one of the two ingroup species, as well as the fact that in any given reconstruction, the calculated intron density is limited to the genes involved in the reconstruction. With similar caveats, the low ancestral minor intron density we report in Zoopagomycota is notable as that group contains *Basidiobolus meristosporus*, which has the highest minor intron density so far discovered in fungi (0.554%). Overall, these results paint a picture of ancestral minor intron complements as generally analogous to those of minor-intron-rich extant species, and highlight the volatile nature of minor intron loss dynamics throughout eukaryotic diversity. It would be exciting to have these results expanded upon once phylogenetic uncertainty has been reduced throughout the tree and an even greater number of diverse genomes are available for analysis.

### Unprecedented minor intron density in the fungus *Rhizophagus irregularis*

**Figure 9.**
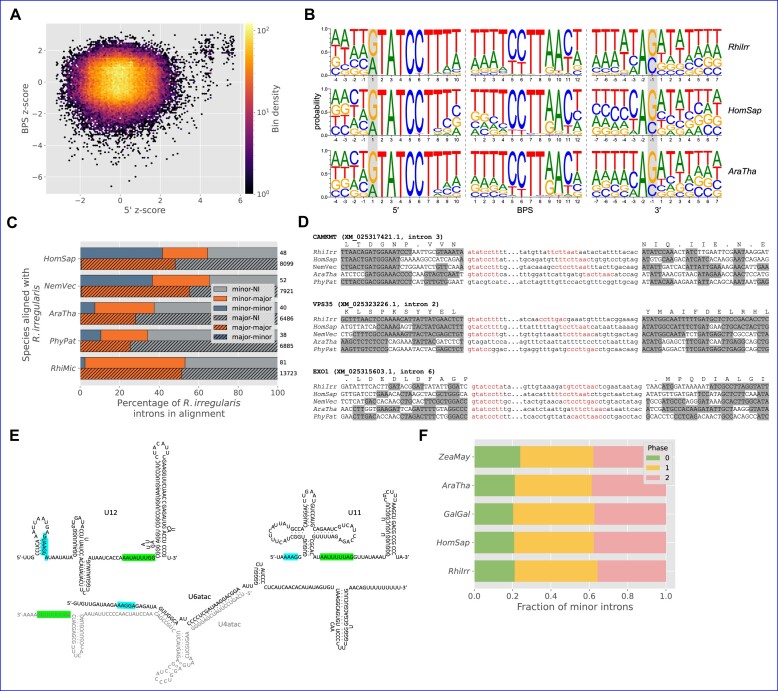
Evidence of minor introns and splicing machinery in *Rhizophagus irregularis*. (**A**) BPS versus 5′SS scores for annotated introns in *Rhizophagus*, showing the expected cloud of introns with minor-intron-like 5′SS and BPS scores in the first quadrant. (**B**) Comparison of minor intron sequence motifs in *Rhizophagus*, human and *Arabidopsis*. (**C**) Conservation states of *Rhizophagus* minor and major introns in different species. NI = ‘No Intron’. (**D**) Examples of *Rhizophagus* minor introns in conserved alignments with minor introns in other species. (**E**) The four minor snRNAs U11, U12, U4atac and U6atac found in *Rhizophagus*. SM binding sites are in green; sequences predicted to basepair with intronic motifs are in cyan. **(F)** Comparison of minor intron phase distributions in different species including *Rhizophagus*, showing the expected bias away from phase 0 in all species. Species abbreviations are as follow: HomSap: *Homo sapiens*, NemVec: *Nematostella vectensis*, AraTha: *Arabidopsis thaliana*, PhyPat: *Physcomitrium patens*, RhiMic: *Rhizopus microsporus*, ZeaMay: *Zea mays*, GalGal: *Gallus gallus*.

In our broad survey of eukaryotic species, we found a large number of putative minor introns in the mycorrhizal fungus *Rhizophagus irregularis*, a member of the Glomeromycota group of fungi. There is clear correspondence between minor-versus-major spliceosomal sequence characteristics in the two primary differentiating parts of the introns, namely the 5′ splice site and the 3′ branchpoint structure (Figure 9A), and consensus sequence features closely follow those previously found in animals and plants (Figure 9B). A subset of minor introns were found at conserved gene positions with minor introns in other fungi, animals and plants (Figure 9C,D), further increasing our confidence that these introns represent bona fide minor spliceosomal introns. Searches of the genome provided additional evidence for the presence of many minor spliceosome-specific proteins as well as all four minor spliceosome-specific non-coding RNAs (U11, U12, U4atac and U6atac) (Figure 9E). As has been shown previously in animals and plants, we found a distinctive distribution of intron phase (position at which introns interrupt the coding codon series, whether between codons (phase 0) or after the first or second nucleotide of a codon (phase 1 and 2, respectively): whereas major intron phases typically follow the pattern 0 > 1 > 2, minor introns in *Rhizophagus* followed the pattern described for minor intron phase in animals and plants (1 > 2 > 0; (19,36)) (Figure 9F). In total, we predict that 205 introns in *Rhizophagus* are minor-type (0.272% of 75,377 annotated introns), orders of magnitude higher than in other fungal species previously reported to contain minor introns (≈4 in *Rhizopus oryzae* and ≈20 in *Phycomyces blakesleeanus*) (34).

### No evidence for increased minor splicing in proliferating cells of *Rhizophagus*

**Figure 10.**
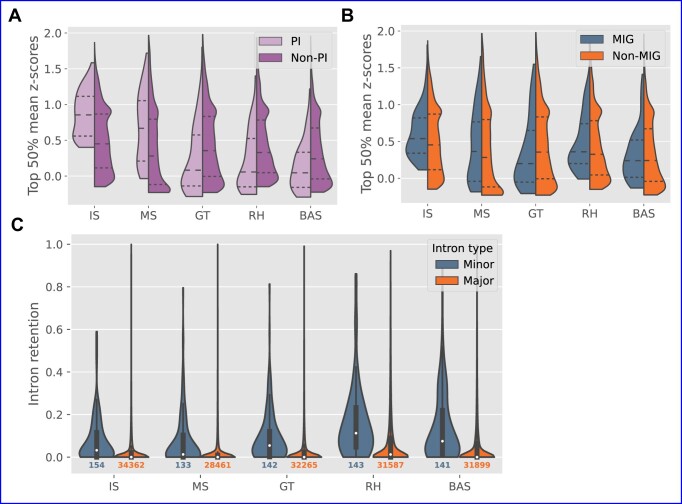
**Gene expression and intron retention comparisons across cell types in Rhizophagus irregularis**. (**A**) Comparison of expression of proliferation-index genes (PI, light purple) and all other genes (non-PI, dark purple) across cell types, *n* = 70 PI and *n* = 9276 non-PI in each cell type. (**B**) As in (A), but for minor intron-containing genes (MIGs) compared to non-MIGs; *n* = 96 MIG and *n* = 9249 non-MIG for each cell type. (**C**) Intron retention values across cell types for minor (blue, left) and major (orange, right) introns. Cell types are labeled as described in the text.

We next sought to test whether *Rhizophagus*, like animals and plants, upregulates splicing of minor introns in proliferating cells. We used published transcriptomic data from five cell types (four replicates each), and assessed likely proliferation profiles of the six cell types using the previously published proliferation index (PI) approach (64). Briefly, we first identified putative orthologs of genes known to be associated with cell proliferation in humans. For each such putative PI ortholog, z-scores were calculated for all 20 samples, and those z-scores were then used for comparison across cell types as well as for comparisons within cell types between putative PI orthologs and other genes. This allowed us to calculate relative proliferation scores for all five cell types. While 4/5 cell types showed similar PI values, one cell type, immature spores, showed substantially and significantly higher values (Figure 10A), a pattern that also held when we look at the more straightforward metric of adjusted FPKM values (Supplementary Figure S4). This overall significance notwithstanding, it should be noted that only a small fraction of genes included in the PI individually showed significant differences in expression between cell types. In addition, we noted that many non-PI genes are also overexpressed in immature spores relative to other cell types; while one interpretation of this result is that it reflects generally more active gene expression in proliferating cells, it does provide a caveat for the overall strength of the observed difference.

We then tested the association between markers of minor spliceosomal activity and these proliferation scores. We first looked for systematic differences in aggregate gene expression of MIGs between cell types with different proliferation scores, using various approaches. First, using the same z-score based approach as for the proliferation score (though with MIGs instead of putative PI orthologs), we found that MIGs were in fact more highly expressed in cell types with higher proliferation scores (Figure 10B). On the other hand, we found that very few MIGs reached significant levels of differential expression, and were in fact underrepresented among genes that showed significant differential expression in multiple comparisons between cell types of different proliferation index scores (e.g., 4.2% of minor intron-containing genes compared to 21.9% of other genes in the IS-MS comparison). In total, these results suggest that expression of MIGs shows a detectable but only moderate association with proliferation index in *Rhizophagus*, in contrast to the robust results previously observed in humans.

We next compared the efficiency of minor intron splicing between cell types. Contrary to our hypothesis that minor splicing would be more active in proliferating cells, we found that minor intron retention was in fact significantly (though only modestly) higher in proliferating cells (Figure 10C). This result held whether we used z-score-based metrics or the intron retention values themselves, and whether we used splicing efficiency or intron retention as our metric. We also assessed expression of the minor splicing machinery itself (i.e. the known components of the minor spliceosome). In comparisons between immature spores and other cell types, no component individually showed higher expression, however collectively the machinery was 3.5x more highly expressed in immature spores than other cell types, reaching significance when considered collectively. However, the major spliceosomal machinery also showed a similar pattern (with 5x higher expression), and as such it seems that lower expression of the minor splicing machinery could be part of a larger pattern of up/regulation of core molecular functions in proliferating/quiescent cells.

The observed association between minor intron splicing and cell proliferation in animals resonates with the long-standing finding that minor introns are overrepresented in genes involved in core cellular processes. Given that minor intron splicing in *Rhizophagus* does not appear to be associated with cell proliferation, we probed these patterns more deeply.

Gene ontology analysis of *Rhizophagus* MIGs revealed a curious pattern in which GO results were highly dependent on the control dataset used. Because of the dearth of *Rhizophagus* functional annotations, GO analyses were necessarily run by identifying human orthologs of *Rhizophagus* MIGs. When GO analysis was run on these orthologs as a subset of all human genes, a number of overrepresented functional categories were found, in large part mirroring results for humans. However, we realized that there is a potential bias in this analysis: all human genes present in the *Rhizophagus* MIG ortholog set have *Rhizophagus* orthologs, thus excluding most human genes (only 14%, 3190/23257, had identified *Rhizophagus* orthologs), and in particular animal-specific genes. Remarkably, when we limited our GO analysis control group to human genes with *Rhizophagus* orthologs, we found much less functional overrepresentation (Table 3).

**Table 3.** GO term enrichment for MIGs in *Rhizophagus* (RiMIGs), compared to all human-*Rhizophagus* orthologs (Hs-Ri)

GO term	Hs-Ri	RiMIGs	E	O/U	FE	FDR
Vesicle-mediated transport (GO:0016192)	288	33	12.82	+	2.57	1.16E-02
Intracellular transport (GO:0046907)	434	39	19.32	+	2.02	3.00E-02
Establishment of localization in cell (GO:0051649)	476	42	21.19	+	1.98	2.84E-02
Small molecule metabolic process (GO:0044281)	549	5	24.44	–	.20	6.81E-03
Carboxylic acid metabolic process (GO:0019752)	309	1	13.75	–	.07	3.41E-02
Oxoacid metabolic process (GO:0043436)	314	1	13.98	–	.07	4.43E-02
Organic acid metabolic process (GO:0006082)	319	1	14.20	–	.07	3.34E-02

E: expected, O/U: over/under, FE: fold enrichment, FDR: false-discovery rate.

Notably, a similar concern applies to human MIGs in general: because nearly all human minor introns are quite old, human MIGs are commensurately old, which could drive functional correlations given known differences in functional categories between genes of different ages. Indeed, when we performed a GO analysis of human MIGs with *Rhizophagus* orthologs, limiting the reference set to human genes with *Rhizophagus* orthologs (a rough surrogate for gene age given that, unlike baker’s yeast, *Rhizophagus* may not have lost many ancestral genes (94)), we found a much lower degree of functional enrichment (Supplementary Table S2). These results support the conclusion that the long-standing result that minor introns are functionally overrepresented in core cellular processes may be largely explained by the fact that minor introns fall primarily in evolutionarily older genes, which are overrepresented in core cellular functions. Interestingly, when we compared all human MIGs (given that minor intron presence strongly suggests that a gene is ancient) to human genes with *Rhizophagus* orthologs, we did see a significant number of overrepresented functional categories (https://doi.org/10.6084/m9.figshare.20483841). It is not entirely clear why all MIGs, but not MIGs with *Rhizophagus* orthologs, show substantial functional differences relative to all genes with *Rhizophagus* orthologs. Insofar as MIGs are ancient genes, MIGs without *Rhizophagus* orthologs likely represent losses in fungi; gene losses are likely to be functionally biased, perhaps explaining the observed pattern.

## Discussion

### An expanded view of minor intron diversity

Over the past decade (after many, if not all, of the most prominent papers examining minor intron diversity were published), there has been a marked increase in the number of annotated genomes publicly available for bioinformatic analysis. Ten years ago, for example, NCBI had annotated fewer than 60 genomes—it now lists over 1000, and that is counting only annotations performed by NCBI itself. The breadth of data now available has enabled us to undertake a much more sweeping assessment of minor intron diversity than has ever been possible before, uncovering a wide variety of both novel and confirmatory information about minor intron dynamics across the eukaryotic tree.

We have shown for the first time the presence of substantial numbers of minor introns as well as minor spliceosomal snRNAs in a variety of lineages previously thought to be lacking them, including green algae, fungi and stramenopiles (see Minor intron enrichment section), as well as a number of cases of potentially complete minor intron loss in both Diptera and Crustacea (Minor intron depletion). In addition, we have described findings contradicting a number of longstanding results in the minor intron literature: compared to major introns, minor introns are almost universally less-well conserved (Minor introns have lower average conservation than major introns), and do not seem—outside of plants—to be especially 5′ biased (Positional biases of major and minor introns). Furthermore, we have highlighted underappreciated differences between MIGs and other genes, namely that MIGs are on average longer and more intron-dense than non-MIGs (Minor intron-containing genes are longer and more intron-rich than other genes), differences which are not simply explained by differences in gene age (Supplementary Figure S3). We have also compiled the largest-scale data on non-canonical minor intron boundaries and minor intron lengths to date (Non-canonical minor intron splice boundaries, Comparison of minor and major intron lengths), examined variation in the process of intron loss across minor and major introns (Minor intron loss versus conversion), and derived for the first time estimates of ancestral minor intron densities for various eukaryotic clades (Reconstruction of ancestral minor intron densities). We have also made use of our discovery of large numbers of minor introns in the fungus *Rhizophagus irregularis* to evaluate the origins and potential confounding factors of previously-described functional biases in minor introns through the lens of neutral evolution (Unprecedented minor intron density in the fungus *Rhizophagus irregularis*). Finally, we have developed an updated, publicly-available database containing minor intron information from more than 1500 species (https://www.introns.info), which we hope will serve as a useful resource for future investigations into the many remaining questions related to minor introns and their evolution.

Although we have been as careful as possible in curating the data for this study, as with most computational endeavors of this scale there is bound to be some amount of noise, especially given our reliance on existing gene annotations derived from heterogeneous pipelines. One persistent issue in bioinformatic analyses of minor introns is the lack of a gold standard, empirically-verified set of minor intron sequences. While comparative genomics can do a great deal of heavy lifting in this regard, it is often a time-consuming process at scale and the field in general would benefit greatly from a ground-truth set of minor introns. We look forward to this type of data—based upon minor spliceosome profiling or another similarly-empirical method—being used to improve the accuracy of minor intron identification and as a result, furthering our understanding of minor introns and their evolutionary dynamics.

### A complex history of minor intron evolution

These results underscore a complex history of minor intron evolution. We greatly expand the number of major eukaryotic groups known to contain minor introns including multiple unicellular lineages, highlighting the punctate distribution of minor introns. We show that multiple distantly-related lineages of fungi contain minor intron densities comparable to animals and plants. However, these three groups show dramatically different patterns of minor intron distribution. At one extreme, land plants show a high degree of minor intron stasis, with similar minor intron densities across nearly all studied species. At the other extreme, fungi exhibit a wide diversity, with high minor intron densities in multiple lineages, greatly reduced numbers in multiple others, and complete absence from the globally dominant group Dikarya. Animals are somewhat intermediate, with minor intron presence in nearly all groups, but a range from very high to very low densities, and even multiple independent complete losses of minor introns. Of particular interest is the case of Dipterans, which exhibit massive reduction across the group, and yet almost no cases of complete loss. If is of great interest why these few minor introns have been so strongly retained across this clade. The diversity of minor intron conservation is also observed in terms of rates, with remarkable stasis in some groups (particularly vertebrates) contrasting with rapid turnover within single genera (e.g., *Blastocystis*, 92% (11/12) of minor introns in alignments between *Blastocystis sp. subtype 1* and *Blastocystis hominis* lost in *B. hominis*). We also document the remarkable diversity of the mechanisms by which minor introns are lost from genomes, ranging from almost exclusively deletion in certain lineages to primarily conversion in others.

Our ancestral reconstructions suggest that ancestors of major groups (plants, animals, fungi) likely had modern densities comparable to the most minor intron-rich modern organisms (aside from the exceptional case of *Physarum* (20)). Coupled with very little evidence for *de novo* minor intron creation, this suggests a portrait in which modern organisms are largely minor intron-rich insofar as they have retained ancestral minor intron complements. The implied portrait contrasts with notions of multicellular organisms as ‘highly-evolved’; rather, higher minor intron complements largely reflect lack of evolutionary change. The same contrast applies to within-kingdom comparisons: in particular, the animal lineages that have lost their minor spliceosomal systems (nematodes, myxozoa, oikopleuridae, tardigrades) have all been found to be generally fast-evolving at the genome level, including in terms of ancestral loss of spliceosomal introns overall ((81,95,96), GEL and SWR unpublished data).

### Many features of minor intron evolution are consistent with neutral evolution

Attempts to make sense of the minor introns have generally alternatively argued they are functionally important or deleterious. Arguments for minor introns’ importance have noted their over-representation in genes with certain functions, associations of minor splicing with cell differentiation including apparent master regulatory roles, and one influential study finding greater evolutionary conservation of minor introns. Arguments that minor introns are deleterious tend to invoke their generally lower efficiency of splicing in addition to the complications and costs associated with maintaining two separate spliceosomal machineries.

Our results are not supportive of either of these perspectives as a general explanation for minor introns across eukaryotes. First, we show that apparent functional biases among minor intron-containing genes may be largely explained by minor introns’ bias towards ancient genes: because most minor introns are old, most MIGs are also old, and core cellular processes are over-represented among old genes. This suggests that minor intron distributions across genes could simply reflect largely unbiased minor intron gain in ancestral genomes, followed by a lack of minor gain in more recent evolutionary time. Second, from our preliminary data in the minor intron-rich fungus *Rhizophagus irregularis* it does not appear that minor splicing is associated with cell proliferation in this species, suggesting that such an association may be specific to certain lineages and thus not capable of explaining general features of minor introns across eukaryotes. Third, we find a remarkably constant (though slight) trend for lesser, not greater, conservation of minor introns compared to major introns. Interestingly, we find very similar rates of intron loss by genomic deletion for minor and major introns, suggesting that minor introns’ somewhat lower overall evolutionary conservation reflects minor introns’ ‘extra’ mechanism of loss through conversion to major introns. Our finding of similar rates of minor and major intron deletion is also not as predicted if minor introns are deleterious relative to major introns. Notably, this lack of an excess of minor intron loss is also observed in the lineages experiencing high degrees of minor-to-major conversion, which represent the best candidates for lineages in which minor introns might be deleterious. In total, then, our results suggest that neutral processes can explain much of the observed minor intron pattern across eukaryotes. This is not to say that all minor intron evolution is neutral, particularly in light of important cases of regulated and alternative splicing of minor introns; however, it may be the case that neutral processes govern most minor introns under most circumstances, and thus underlie observed patterns across both genomes and lineages.

### Secondary recruitment of ancient machineries for cell cycle regulation

Prior to the current work, we perceived a chicken and egg problem of functional biases among MIGs (21,31) and control of cell proliferation by regulation of minor splicing (24,27,28): that is, how could the regulatory control evolve without the functional bias, by why would the functional bias evolve without the regulatory function? We thus sought to illuminate this question by studying a third minor intron-rich lineage. The current findings that the observed functional biases appear to be largely explained by minor introns’ bias towards older genes, and older genes bias towards core cellular functions, suggest an answer. Thus, functional biases could have initially evolved due to these gene age biases, and this functional bias could then have secondarily been recruited to regulate cell proliferation in animals in plants.

While this scenario makes sense schematically, is remains a remarkable contention that decreased minor splicing could evolve a function in cell regulation; insofar as MIGs represent a quasi-random subset of ancient genes, it seems likely that a global reduction in minor splicing would have a wide variety of impacts, many of them likely costly. Thus how failure to process a quasi-randomly chosen set of ancestral genes could evolve as a regulatory mechanism remains puzzling, and will require additional work across diverse minor intron-containing lineages.

Our results do not support the emerging dominant hypothesis for the existence of minor introns, namely that minor introns provide a means for regulation of cell cycle progression. The reported lack of cell cycle-regulated minor intron splicing in fungi suggests that this association is not a general phenomenon, correspondingly weakening the hypothesis that such a function could explain the persistence of minor introns across eukaryotes generally. However, given the possibility that it may be fungi that are atypical, having secondarily lost this function, discovery and study of additional minor intron-rich lineages is a priority, as is development and testing of alternative hypotheses for the origins and functional biases of minor intron-containing genes.

### Limitations of the *Rhizophagus* analysis

Possible caveats of this analysis arise from two surrogates that we have employed. First, to assess cell cycle activity/proliferation of cell types, we have used orthologs of human genes associated with proliferation. The possibility of turnover of gene expression patterns raises the concern that these genes are not an appropriate gene set to assess proliferation. Indeed, while clear statistical differences in proliferation are seen when PI genes are viewed collectively, only a small fraction ($\approx 5-10\%$) individually show significantly different expression between cell types. However, similar comparisons with model fungi attest to generally good conservation of genes’ association with proliferation, consistent with an ancient core of cell cycle regulation. Second, we have used available transcriptomic data not specifically generated for the purposes of comparing proliferation, potentially leading to noise in the data. However, the general pattern observed, in which developing spores show the highest proliferation index, mirrors intuitive expectations, suggesting that our proliferation scores are capturing at least some of the relevant biological phenomena. Testing of transcriptomic effects of direct manipulations of cell cycle would be very useful to confirm (or refute) our results.

## Supplementary Material

gkad797_Supplemental_File

## Data Availability

Metadata for all species in this paper identified as having minor introns, including intron classification data for all annotated introns used in our analyses, is available via an interactive online database located at https://www.introns.info. The underlying database file has been archived in Dryad at https://doi.org/10.6071/M36Q39. Plain text data for Figure S1 is archived on FigShare at https://doi.org/10.6084/m9.figshare.20483655. Additional versions of certain figures, including a linear version of Figure S1 and interactive versions of the plots in Figure 7 are available at https://www.github.com/glarue/minor_introns. The following permanent GitHub repository archives have been created: github.com/glarue/minor_introns at http://doi.org/10.5281/zenodo.8355612, github.com/glarue/cdseq at https://doi.org/10.5281/zenodo.8355554, and github.com/glarue/reciprologs at http://doi.org/10.5281/zenodo.8355558.

## References

[1] Will C.L. , Lührmann R. Spliceosome structure and function. Cold Spring Harb. Perspect. Biol. 2011; 3:a003707. 21441581 10.1101/cshperspect.a003707 PMC3119917

[2] Matera A.G. , Wang Z. A day in the life of the spliceosome. Nat. Rev. Mol. Cell Biol. 2014; 15:108–121. 24452469 10.1038/nrm3742 PMC4060434

[3] Jurica M.S. Detailed close-ups and the big picture of spliceosomes. Curr. Opin. Struct. Biol. 2008; 18:315–320. 18550358 10.1016/j.sbi.2008.05.005 PMC2474778

[4] Jeffreys A.J. , Flavell R.A. The rabbit beta-globin gene contains a large large insert in the coding sequence. Cell. 1977; 12:1097–1108. 597859 10.1016/0092-8674(77)90172-6

[5] Gilbert W. Why genes in pieces?. Nature. 1978; 271:501. 622185 10.1038/271501a0

[6] Brack C. , Tonegawa S. Variable and constant parts of the immunoglobulin light chain gene of a mouse myeloma cell are 1250 nontranslated bases apart. Proc. Natl. Acad. Sci. U.S.A. 1977; 74:5652–5656. 414223 10.1073/pnas.74.12.5652 PMC431846

[7] Breathnach R. , Chambon P. Organization and expression of eucaryotic split genes coding for proteins. Annu. Rev. Biochem. 1981; 50:349–383. 6791577 10.1146/annurev.bi.50.070181.002025

[8] Padgett R.A. , Grabowski P.J., Konarska M.M., Seiler S., Sharp P.A. Splicing of messenger RNA precursors. Annu. Rev. Biochem. 1986; 55:1119–1150. 2943217 10.1146/annurev.bi.55.070186.005351

[9] Mount S.M. A catalogue of splice junction sequences. Nucleic Acids Res. 1982; 10:459–472. 7063411 10.1093/nar/10.2.459 PMC326150

[10] Jackson I.J. A reappraisal of non-consensus mRNA splice sites. Nucleic Acids Res. 1991; 19:3795–3798. 1713664 10.1093/nar/19.14.3795 PMC328465

[11] Hall S.L. , Padgett R.A. Conserved sequences in a class of rare eukaryotic nuclear introns with non-consensus splice sites. J. Mol. Biol. 1994; 239:357–365. 8201617 10.1006/jmbi.1994.1377

[12] Tarn W.Y. , Steitz J.A. A novel spliceosome containing U11, U12, and U5 snRNPs excises a minor class (AT-AC) intron in vitro. Cell. 1996; 84:801–811. 8625417 10.1016/s0092-8674(00)81057-0

[13] Tarn W.Y. , Steitz J.A. Highly diverged U4 and U6 small nuclear RNAs required for splicing rare AT-AC introns. Science. 1996; 273:1824–1832. 8791582 10.1126/science.273.5283.1824

[14] Russell A.G. , Charette J.M., Spencer D.F., Gray M.W. An early evolutionary origin for the minor spliceosome. Nature. 2006; 443:863–866. 17051219 10.1038/nature05228

[15] Frey K. , Pucker B. Animal, fungi, and plant genome sequences harbor different non-canonical splice sites. Cells. 2020; 9:458. 32085510 10.3390/cells9020458 PMC7072748

[16] Burset M. , Seledtsov I.A., Solovyev V.V. Analysis of canonical and non-canonical splice sites in mammalian genomes. Nucleic Acids Res. 2000; 28:4364–4375. 11058137 10.1093/nar/28.21.4364 PMC113136

[17] Pucker B. , Brockington S.F. Genome-wide analyses supported by RNA-Seq reveal non-canonical splice sites in plant genomes. BMC Genomics. 2018; 19:980. 30594132 10.1186/s12864-018-5360-z PMC6310983

[18] Sibley C.R. , Blazquez L., Ule J. Lessons from non-canonical splicing. Nat. Rev. Genet. 2016; 17:407–421. 27240813 10.1038/nrg.2016.46 PMC5154377

[19] Moyer D.C. , Larue G.E., Hershberger C.E., Roy S.W., Padgett R.A. Comprehensive database and evolutionary dynamics of U12-type introns. Nucleic Acids Res. 2020; 48:7066–7078. 32484558 10.1093/nar/gkaa464 PMC7367187

[20] Larue G.E. , Eliáš M., Roy S.W. Expansion and transformation of the minor spliceosomal system in the slime mold *Physarum polycephalum*. Curr. Biol. 2021; 31:3125–3131. 34015249 10.1016/j.cub.2021.04.050

[21] Lin C.-F. , Mount S.M., Jarmołowski A., Makałowski W. Evolutionary dynamics of U12-type spliceosomal introns. BMC Evol. Biol. 2010; 10:47. 20163699 10.1186/1471-2148-10-47 PMC2831892

[22] Alioto T.S. U12DB: a database of orthologous U12-type spliceosomal introns. Nucleic Acids Res. 2007; 35:D110–D115. 17082203 10.1093/nar/gkl796 PMC1635337

[23] Dávila López M. , Rosenblad M.A., Samuelsson T. Computational screen for spliceosomal RNA genes aids in defining the phylogenetic distribution of major and minor spliceosomal components. Nucleic Acids Res. 2008; 36:3001–3010. 18390578 10.1093/nar/gkn142 PMC2396436

[24] Gault C.M. , Martin F., Mei W., Bai F., Black J.B., Barbazuk W.B., Settles A.M. Aberrant splicing in maize rough endosperm3 reveals a conserved role for U12 splicing in eukaryotic multicellular development. Proc. Natl. Acad. Sci. U.S.A. 2017; 114:E2195–E2204. 28242684 10.1073/pnas.1616173114 PMC5358371

[25] Doggett K. , Williams B.B., Markmiller S., Geng F.-S., Coates J., Mieruszynski S., Ernst M., Thomas T., Heath J.K. Early developmental arrest and impaired gastrointestinal homeostasis in U12-dependent splicing-defective Rnpc3-deficient mice. RNA. 2018; 24:1856–1870. 30254136 10.1261/rna.068221.118 PMC6239176

[26] König H. , Matter N., Bader R., Thiele W., Müller F. Splicing segregation: the minor spliceosome acts outside the nucleus and controls cell proliferation. Cell. 2007; 131:718–729. 18022366 10.1016/j.cell.2007.09.043

[27] Meinke S. , Goldammer G., Weber A.I., Tarabykin V., Neumann A., Preussner M., Heyd F. Srsf10 and the minor spliceosome control tissue-specific and dynamic SR protein expression. eLife. 2020; 9:e56075. 32338600 10.7554/eLife.56075 PMC7244321

[28] Bai F. , Corll J., Shodja D.N., Davenport R., Feng G., Mudunkothge J., Brigolin C.J., Martin F., Spielbauer G., Tseung C.-W. et al. RNA binding motif protein 48 Is required for U12 splicing and maize endosperm differentiation. Plant Cell. 2019; 31:715–733. 30760564 10.1105/tpc.18.00754 PMC6482629

[29] Najle S.R. , Ruiz-Trillo I. The protistan origins of animal cell differentiation. Origin and Evolution of Metazoan Cell Types. 2021; CRC Press 13–26.

[30] Brunet T. , Larson B.T., Linden T.A., Vermeij M. J.A., McDonald K., King N. Light-regulated collective contractility in a multicellular choanoflagellate. Science. 2019; 366:326–334. 31624206 10.1126/science.aay2346

[31] Burge C.B. , Padgett R.A., Sharp P.A. Evolutionary fates and origins of U12-type introns. Mol. Cell. 1998; 2:773–785. 9885565 10.1016/s1097-2765(00)80292-0

[32] Baumgartner M. , Olthof A.M., Aquino G.S., Hyatt K.C., Lemoine C., Drake K., Sturrock N., Nguyen N., Al Seesi S., Kanadia R.N. Minor spliceosome inactivation causes microcephaly, owing to cell cycle defects and death of self-amplifying radial glial cells. Development. 2018; 145:17. 10.1242/dev.166322 PMC6141777 30093551

[33] Sharp P.A. , Burge C.B. Classification of introns: U2-type or U12-type. Cell. 1997; 91:875–879. 9428511 10.1016/s0092-8674(00)80479-1

[34] Bartschat S. , Samuelsson T. U12 type introns were lost at multiple occasions during evolution. BMC Genomics. 2010; 11:106. 20149226 10.1186/1471-2164-11-106 PMC2846911

[35] Sheth N. , Roca X., Hastings M.L., Roeder T., Krainer A.R., Sachidanandam R. Comprehensive splice-site analysis using comparative genomics. Nucleic Acids Res. 2006; 34:3955–3967. 16914448 10.1093/nar/gkl556 PMC1557818

[36] Levine A. , Durbin R. A computational scan for U12-dependent introns in the human genome sequence. Nucleic Acids Res. 2001; 29:4006–4013. 11574683 10.1093/nar/29.19.4006 PMC60238

[37] Federhen S. The NCBI Taxonomy database. Nucleic Acids Res. 2012; 40:D136–D143. 22139910 10.1093/nar/gkr1178 PMC3245000

[38] Nawrocki E.P. , Eddy S.R. Infernal 1.1: 100-fold faster RNA homology searches. Bioinformatics. 2013; 29:2933–2935. 24008419 10.1093/bioinformatics/btt509 PMC3810854

[39] Bicknell A.A. , Cenik C., Chua H.N., Roth F.P., Moore M.J. Introns in UTRs: why we should stop ignoring them. Bioessays. 2012; 34:1025–1034. 23108796 10.1002/bies.201200073

[40] Chung B. Y.W. , Simons C., Firth A.E., Brown C.M., Hellens R.P. Effect of 5’UTR introns on gene expression in Arabidopsis thaliana. BMC Genomics. 2006; 7:120. 16712733 10.1186/1471-2164-7-120 PMC1482700

[41] Stark A. , Brennecke J., Bushati N., Russell R.B., Cohen S.M. Animal MicroRNAs confer robustness to gene expression and have a significant impact on 3’UTR evolution. Cell. 2005; 123:1133–1146. 16337999 10.1016/j.cell.2005.11.023

[42] Sharangdhar T. , Sugimoto Y., Heraud-Farlow J., Fernández-Moya S.M., Ehses J., Ruiz de Los Mozos I., Ule J., Kiebler M.A. A retained intron in the 3’-UTR of Calm3 mRNA mediates its Staufen2- and activity-dependent localization to neuronal dendrites. EMBO Rep. 2017; 18:1762–1774. 28765142 10.15252/embr.201744334 PMC5623867

[43] Lu J. , Sivamani E., Azhakanandam K., Samadder P., Li X., Qu R. Gene expression enhancement mediated by the 5’ UTR intron of the rice rubi3 gene varied remarkably among tissues in transgenic rice plants. Mol. Genet. Genomics. 2008; 279:563–572. 18320227 10.1007/s00438-008-0333-6

[44] Roy S.W. , Fedorov A., Gilbert W. Large-scale comparison of intron positions in mammalian genes shows intron loss but no gain. Proc. Natl. Acad. Sci. U.S.A. 2003; 100:7158–7162. 12777620 10.1073/pnas.1232297100 PMC165846

[45] Stoltzfus A. , Logsdon J.M., Palmer J.D., Doolittle W.F. Intron ‘sliding’ and the diversity of intron positions. Proc. Natl. Acad. Sci. U.S.A. 1997; 94:10739–10744. 9380704 10.1073/pnas.94.20.10739 PMC23469

[46] Poverennaya I.V. , Potapova N.A., Spirin S.A. Is there any intron sliding in mammals?. BMC Evol. Biol. 2020; 20:164. 33308147 10.1186/s12862-020-01726-0 PMC7730772

[47] Roy S.W. Intronization, de-intronization and intron sliding are rare in Cryptococcus. BMC Evol. Biol. 2009; 9:192. 19664208 10.1186/1471-2148-9-192 PMC2740785

[48] Sêton Bocco S. , Csűrös M. Splice sites seldom slide: intron evolution in oomycetes. Genome Biol. Evol. 2016; 8:2340–2350. 27412607 10.1093/gbe/evw157 PMC5010894

[49] Simão F.A. , Waterhouse R.M., Ioannidis P., Kriventseva E.V., Zdobnov E.M. BUSCO: assessing genome assembly and annotation completeness with single-copy orthologs. Bioinformatics. 2015; 31:3210–3212. 26059717 10.1093/bioinformatics/btv351

[50] Barrera-Redondo J. , Lotharukpong J.S., Drost H.-G., Coelho S.M. Uncovering gene-family founder events during major evolutionary transitions in animals, plants and fungi using GenEra. Genome Biol. 2023; 24:54. 36964572 10.1186/s13059-023-02895-z PMC10037820

[51] Enright A.J. , Van Dongen S., Ouzounis C.A. An efficient algorithm for large-scale detection of protein families. Nucleic Acids Res. 2002; 30:1575–1584. 11917018 10.1093/nar/30.7.1575 PMC101833

[52] Buchfink B. , Reuter K., Drost H.-G. Sensitive protein alignments at tree-of-life scale using DIAMOND. Nat. Methods. 2021; 18:366–368. 33828273 10.1038/s41592-021-01101-x PMC8026399

[53] Roy S.W. , Gilbert W. Complex early genes. Proc. Natl. Acad. Sci. U.S.A. 2005; 102:1986–1991. 15687506 10.1073/pnas.0408355101 PMC548548

[54] Holland B.R. , Ketelaar-Jones S., O’Mara A.R., Woodhams M.D., Jordan G.J. Accuracy of ancestral state reconstruction for non-neutral traits. Sci. Rep. 2020; 10:7644. 32376845 10.1038/s41598-020-64647-4 PMC7203120

[55] Duchêne S. , Lanfear R. Phylogenetic uncertainty can bias the number of evolutionary transitions estimated from ancestral state reconstruction methods. J. Exp. Zool. B Mol. Dev. Evol. 2015; 324:517–524. 26173578 10.1002/jez.b.22638

[56] Cunningham C.W. Some limitations of ancestral character-state reconstruction when testing evolutionary hypotheses. Syst. Biol. 1999; 48:665–674.

[57] Roy S.W. How common is parallel intron gain? rapid evolution versus independent creation in recently created introns in daphnia. Mol. Biol. Evol. 2016; 33:1902–1906. 27189562 10.1093/molbev/msw091

[58] Sverdlov A.V. , Rogozin I.B., Babenko V.N., Koonin E.V. Conservation versus parallel gains in intron evolution. Nucleic Acids Res. 2005; 33:1741–1748. 15788746 10.1093/nar/gki316 PMC1069513

[59] Carmel L. , Rogozin I.B., Wolf Y.I., Koonin E.V. Patterns of intron gain and conservation in eukaryotic genes. BMC Evol. Biol. 2007; 7:192. 17935625 10.1186/1471-2148-7-192 PMC2151770

[60] Kameoka H. , Maeda T., Okuma N., Kawaguchi M. Structure-specific regulation of nutrient transport and metabolism in arbuscular mycorrhizal fungi. Plant Cell Physiol. 2019; 60:2272–2281. 31241164 10.1093/pcp/pcz122

[61] Patro R. , Duggal G., Love M.I., Irizarry R.A., Kingsford C. Salmon provides fast and bias-aware quantification of transcript expression. Nat. Methods. 2017; 14:417–419. 28263959 10.1038/nmeth.4197 PMC5600148

[62] Soneson C. , Love M.I., Robinson M.D. Differential analyses for RNA-seq: transcript-level estimates improve gene-level inferences. F1000Res. 2015; 4:1521. 26925227 10.12688/f1000research.7563.1 PMC4712774

[63] Love M.I. , Huber W., Anders S. Moderated estimation of fold change and dispersion for RNA-seq data with DESeq2. Genome Biol. 2014; 15:550. 25516281 10.1186/s13059-014-0550-8 PMC4302049

[64] Sandberg R. , Neilson J.R., Sarma A., Sharp P.A., Burge C.B. Proliferating cells express mRNAs with shortened 3’ untranslated regions and fewer microRNA target sites. Science. 2008; 320:1643–1647. 18566288 10.1126/science.1155390 PMC2587246

[65] Middleton R. , Gao D., Thomas A., Singh B., Au A., Wong J.J.-L., Bomane A., Cosson B., Eyras E., Rasko J.E.J. et al. IRFinder: assessing the impact of intron retention on mammalian gene expression. Genome Biol. 2017; 18:51. 28298237 10.1186/s13059-017-1184-4 PMC5353968

[66] Langmead B. Aligning short sequencing reads with Bowtie. Curr. Protoc. Bioinformatics. 2010; Chapter 11:Unit 11.7. 10.1002/0471250953.bi1107s32 PMC3010897 21154709

[67] Camacho C. , Coulouris G., Avagyan V., Ma N., Papadopoulos J., Bealer K., Madden T.L. BLAST+: architecture and applications. BMC Bioinformatics. 2009; 10:421. 20003500 10.1186/1471-2105-10-421 PMC2803857

[68] Letunic I. , Bork P. Interactive Tree Of Life (iTOL) v5: an online tool for phylogenetic tree display and annotation. Nucleic Acids Res. 2021; 49:W293–W296. 33885785 10.1093/nar/gkab301 PMC8265157

[69] Janice J. , Pande A., Weiner J., Lin C.-F., Makałowski W. U12-type spliceosomal introns of Insecta. Int. J. Biol. Sci. 2012; 8:344–352. 22393306 10.7150/ijbs.3933 PMC3291851

[70] Szcześniak M.W. , Kabza M., Pokrzywa R., Gudyś A., Makałowska I. ERISdb: a database of plant splice sites and splicing signals. Plant Cell Physiol. 2013; 54:e10. 23299413 10.1093/pcp/pct001

[71] Turunen J.J. , Niemelä E.H., Verma B., Frilander M.J. The significant other: splicing by the minor spliceosome. Wiley Interdiscip. Rev. RNA. 2013; 4:61–76. 23074130 10.1002/wrna.1141 PMC3584512

[72] Shadwick J. D.L. , Silberman J.D., Spiegel F.W. Variation in the SSUrDNA of the genus protostelium leads to a new phylogenetic understanding of the genus and of the species concept for protostelium mycophaga (Amoebozoa). J. Eukaryot. Microbiol. 2018; 65:331–344. 29044743 10.1111/jeu.12476

[73] Gentekaki E. , Curtis B.A., Stairs C.W., Klimeš V., Eliáš M., Salas-Leiva D.E., Herman E.K., Eme L., Arias M.C., Henrissat B. et al. Extreme genome diversity in the hyper-prevalent parasitic eukaryote Blastocystis. PLoS Biol. 2017; 15:e2003769. 28892507 10.1371/journal.pbio.2003769 PMC5608401

[74] Basu M.K. , Makalowski W., Rogozin I.B., Koonin E.V. U12 intron positions are more strongly conserved between animals and plants than U2 intron positions. Biol. Direct. 2008; 3:19. 18479526 10.1186/1745-6150-3-19 PMC2426677

[75] Cohen N.E. , Shen R., Carmel L. The role of reverse transcriptase in intron gain and loss mechanisms. Mol. Biol. Evol. 2012; 29:179–186. 21804076 10.1093/molbev/msr192

[76] Irimia M. , Roy S.W. Origin of spliceosomal introns and alternative splicing. Cold Spring Harb. Perspect. Biol. 2014; 6:a016071. 24890509 10.1101/cshperspect.a016071 PMC4031966

[77] Dietrich R.C. , Incorvaia R., Padgett R.A. Terminal intron dinucleotide sequences do not distinguish between U2- and U12-dependent introns. Mol. Cell. 1997; 1:151–160. 9659912 10.1016/s1097-2765(00)80016-7

[78] Dietrich R.C. , Fuller J.D., Padgett R.A. A mutational analysis of U12-dependent splice site dinucleotides. RNA. 2005; 11:1430–1440. 16043500 10.1261/rna.7206305 PMC1370826

[79] Frilander M.J. , Steitz J.A. Initial recognition of U12-dependent introns requires both U11/5’ splice-site and U12/branchpoint interactions. Genes Dev. 1999; 13:851–863. 10197985 10.1101/gad.13.7.851 PMC316595

[80] Rogozin I.B. , Carmel L., Csuros M., Koonin E.V. Origin and evolution of spliceosomal introns. Biol. Direct. 2012; 7:11. 22507701 10.1186/1745-6150-7-11 PMC3488318

[81] Mourier T. , Jeffares D.C. Eukaryotic intron loss. Science. 2003; 300:1393. 12775832 10.1126/science.1080559

[82] Lin K. , Zhang D.-Y. The excess of 5’ introns in eukaryotic genomes. Nucleic Acids Res. 2005; 33:6522–6527. 16314314 10.1093/nar/gki970 PMC1292992

[83] Sakurai A. , Fujimori S., Kochiwa H., Kitamura-Abe S., Washio T., Saito R., Carninci P., Hayashizaki Y., Tomita M. On biased distribution of introns in various eukaryotes. Gene. 2002; 300:89–95. 12468090 10.1016/s0378-1119(02)01035-1

[84] Roy S.W. , Gilbert W. The pattern of intron loss. Proc. Natl. Acad. Sci. U.S.A. 2005; 102:713–718. 15642949 10.1073/pnas.0408274102 PMC545554

[85] Roy S.W. , Gilbert W. The evolution of spliceosomal introns: patterns, puzzles and progress. Nat. Rev. Genet. 2006; 7:211–221. 16485020 10.1038/nrg1807

[86] Derr L.K. , Strathern J.N. A role for reverse transcripts in gene conversion. Nature. 1993; 361:170–173. 8380627 10.1038/361170a0

[87] Nguyen H.D. , Yoshihama M., Kenmochi N. Phase distribution of spliceosomal introns: implications for intron origin. BMC Evol. Biol. 2006; 6:69. 16959043 10.1186/1471-2148-6-69 PMC1574350

[88] Long M. , Rosenberg C., Gilbert W. Intron phase correlations and the evolution of the intron/exon structure of genes. Proc. Natl. Acad. Sci. U.S.A. 1995; 92:12495–12499. 8618928 10.1073/pnas.92.26.12495 PMC40384

[89] Burset M. , Seledtsov I.A., Solovyev V.V. SpliceDB: database of canonical and non-canonical mammalian splice sites. Nucleic Acids Res. 2001; 29:255–259. 11125105 10.1093/nar/29.1.255 PMC29840

[90] Parada G.E. , Munita R., Cerda C.A., Gysling K. A comprehensive survey of non-canonical splice sites in the human transcriptome. Nucleic Acids Res. 2014; 42:10564–10578. 25123659 10.1093/nar/gku744 PMC4176328

[91] Wolf Y.I. , Novichkov P.S., Karev G.P., Koonin E.V., Lipman D.J. The universal distribution of evolutionary rates of genes and distinct characteristics of eukaryotic genes of different apparent ages. Proc. Natl. Acad. Sci. U.S.A. 2009; 106:7273–7280. 19351897 10.1073/pnas.0901808106 PMC2666616

[92] Vishnoi A. , Kryazhimskiy S., Bazykin G.A., Hannenhalli S., Plotkin J.B. Young proteins experience more variable selection pressures than old proteins. Genome Res. 2010; 20:1574–1581. 20921233 10.1101/gr.109595.110 PMC2963820

[93] Vinogradov A.E. Intron-genome size relationship on a large evolutionary scale. J. Mol. Evol. 1999; 49:376–384. 10473779 10.1007/pl00006561

[94] Sales-Lee J. , Perry D.S., Bowser B.A., Diedrich J.K., Rao B., Beusch I., Yates J.R. 3rd, Roy S.W., Madhani H.D. Coupling of spliceosome complexity to intron diversity. Curr. Biol. 2021; 31:4898–4910. 34555349 10.1016/j.cub.2021.09.004 PMC8967684

[95] Logsdon J.M. Jr The recent origins of spliceosomal introns revisited. Curr. Opin. Genet. Dev. 1998; 8:637–648. 9914210 10.1016/s0959-437x(98)80031-2

[96] Rogozin I.B. , Wolf Y.I., Sorokin A.V., Mirkin B.G., Koonin E.V. Remarkable interkingdom conservation of intron positions and massive, lineage-specific intron loss and gain in eukaryotic evolution. Curr. Biol. 2003; 13:1512–1517. 12956953 10.1016/s0960-9822(03)00558-x

