# Supplementary material for: Where the minor things are: a pan-eukaryotic survey suggests neutral processes may explain much of minor intron evolution
Source: Nucleic Acids Res. 2023 Oct 11;51(20):10884–908. doi: 10.1093/nar/gkad797 (PMC10639083; doi:10.1093/nar/gkad797)
Supplement: gkad797_Supplemental_File [file gkad797_supplemental_file.pdf]

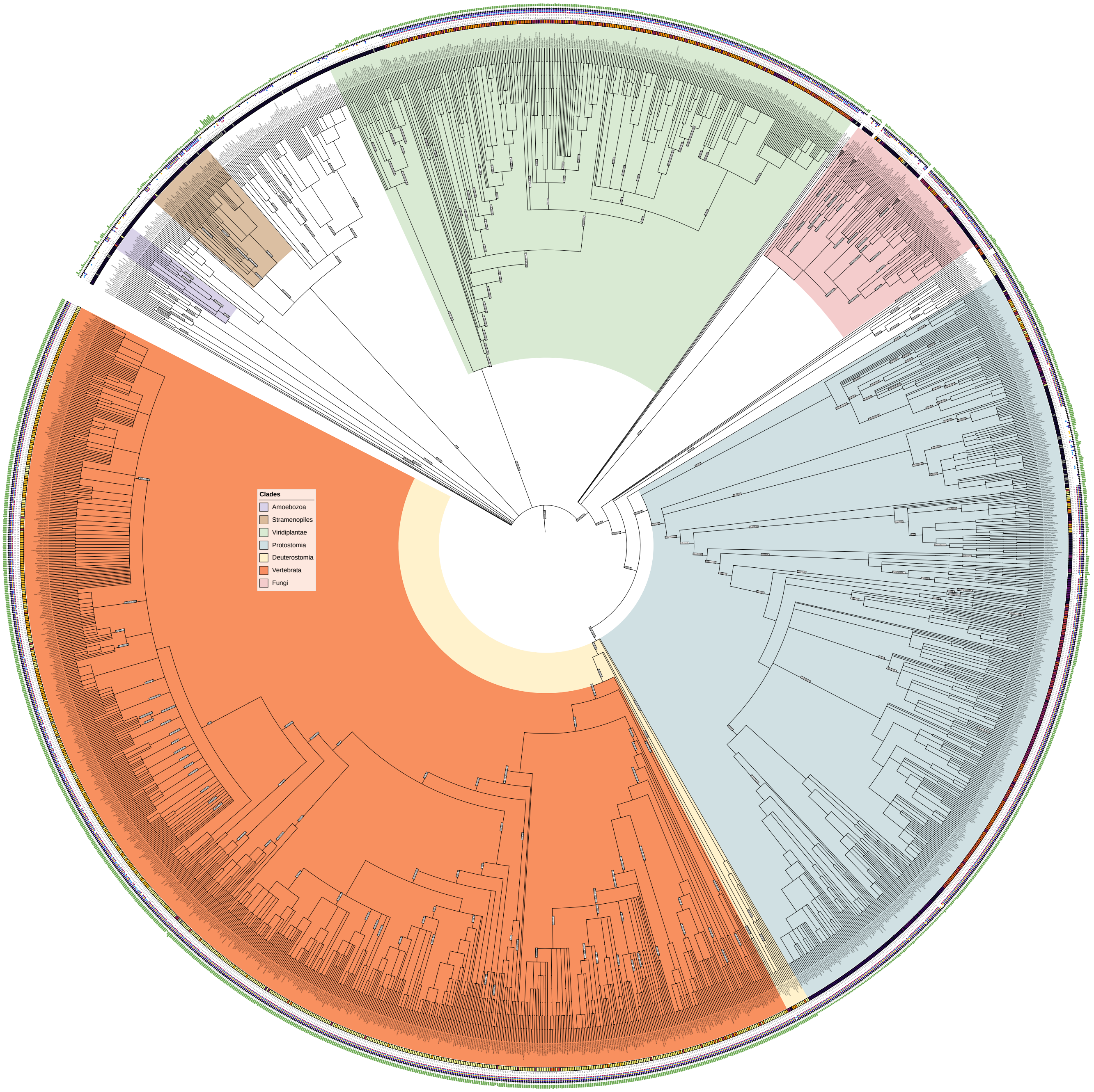

Fig. S1. Minor intron densities for thousands of eukaryotic species. Asterisks following the numbers of putative minor introns (and relative minor intron densities colored gray in the colored strip) indicate less-confident estimates. Other graphical elements as in Figure 1.

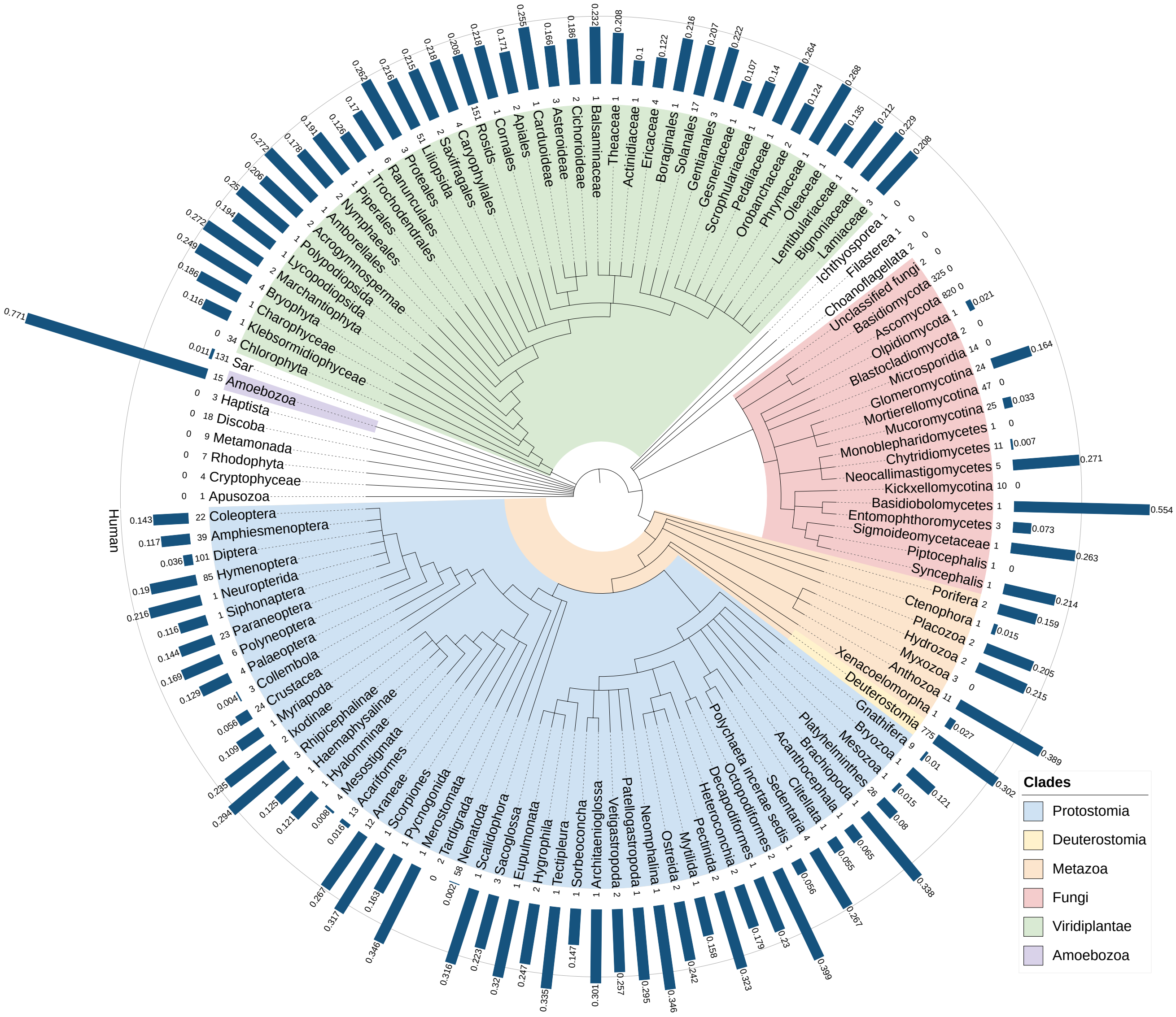

Fig. S2. Average minor intron density (percentage of introns in a genome which are minor type; blue bars) in various eukaryotic clades. Integer numbers following clade names denote number of constituent species. Outer circle corresponds to the minor intron density of the human genome. Taxonomic relationships based upon data from the NCBI Taxonomy Database [37]; figure generated using iTOL [68].

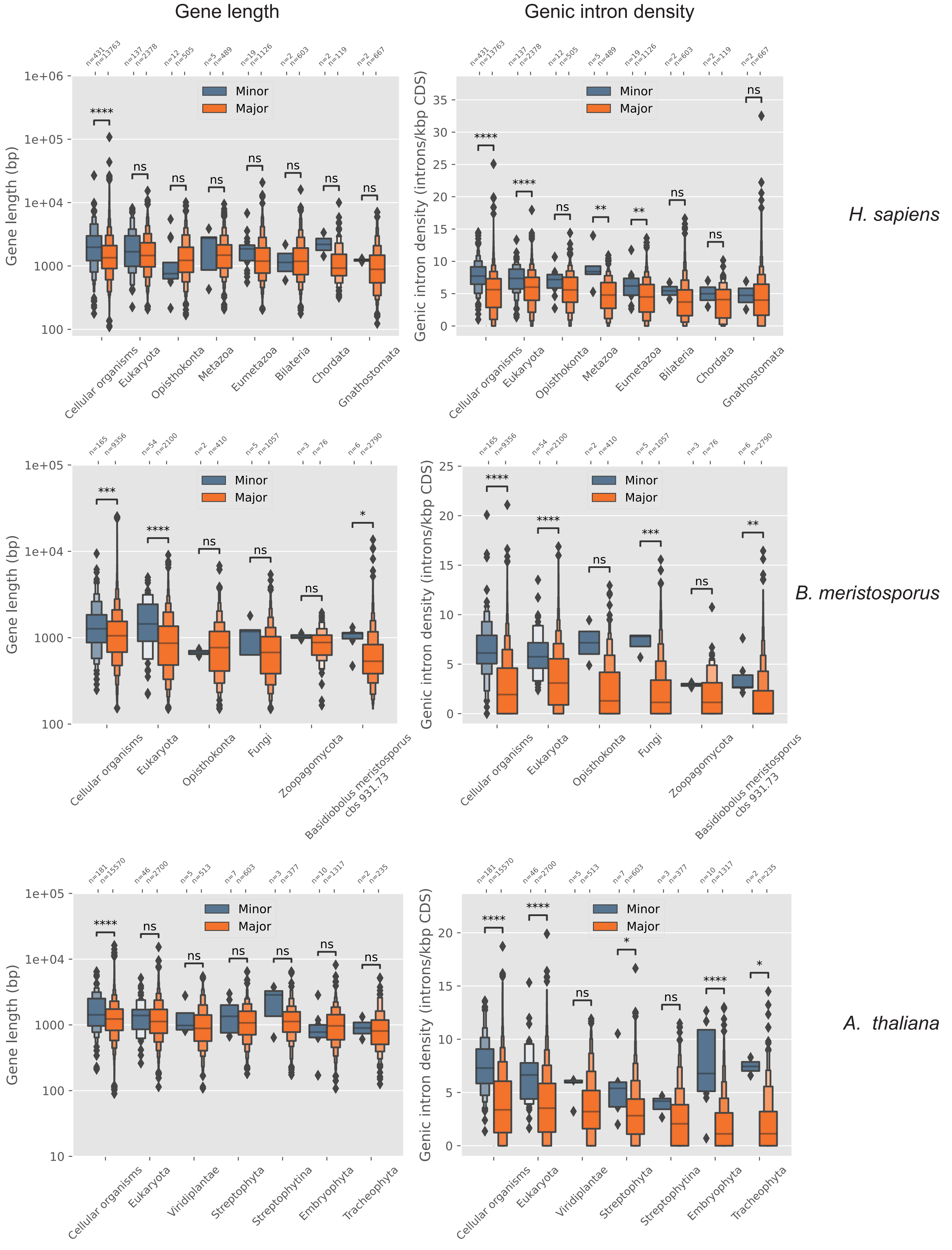

Fig. S3. Comparison of median gene lengths (left) and genic intron densities (right) in MIGs and non-MIGs across different age categories in *Homo sapiens*, *Arabidopsis thaliana* and *Basidiobolus meristosporus*. Numbers of introns of each type in each group indicated at the top of each plot (sample sizes are the same for each age category between plots in each species). Statistically significant differences between minor and major introns are indicated with asterisks (two-tailed Mann-Whitney U test with Benjamini-Hochberg correction; $\star\ p < 0.05$; $\star\star\ p < 0.001$; $\star\star\star\ p < 0.0001$; $\mathtt{ns}$ not significant.

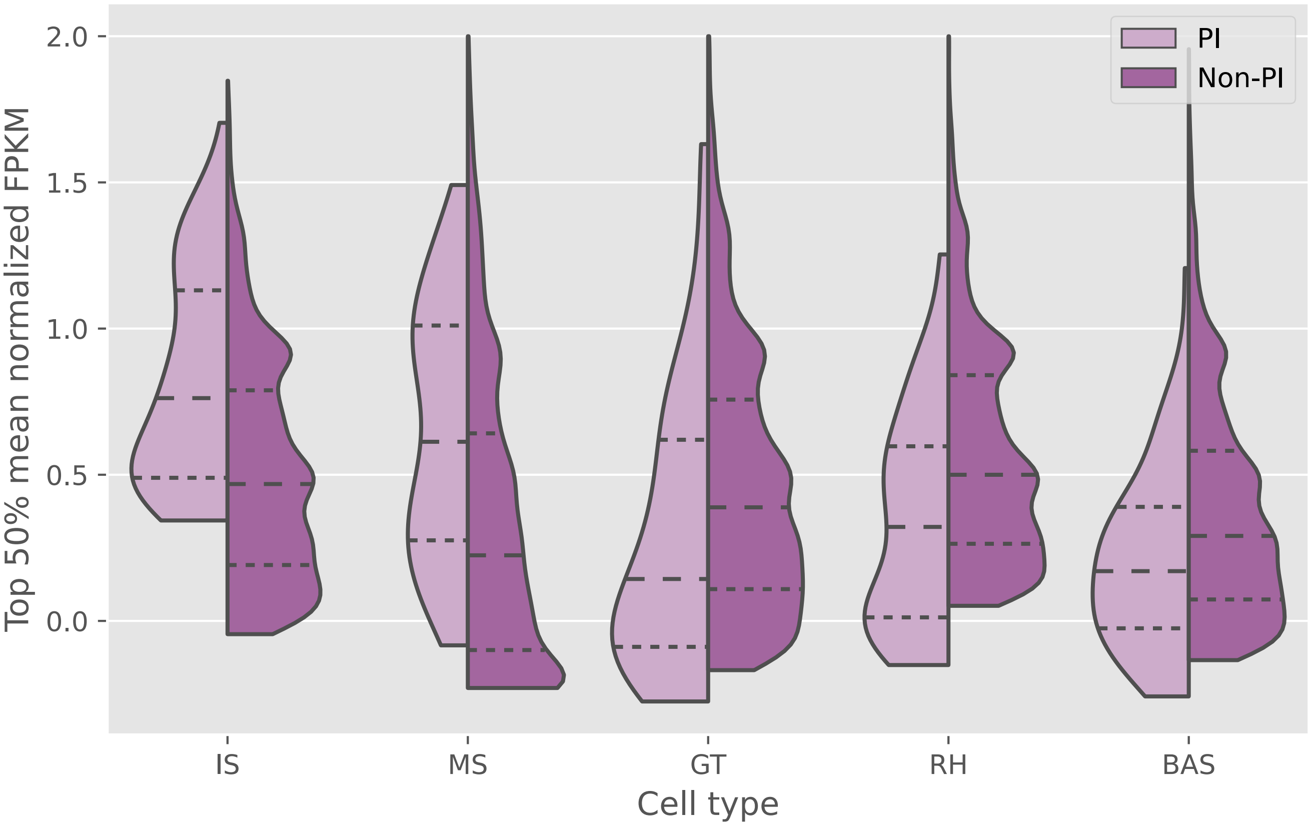

Fig. S4. Comparison of expression (normalized FPKM from `DESeq2`, power-transformed) of proliferation-index genes (PI, light purple) and all other genes (Non-PI, dark purple) across cell types.

Animals			Plants		
Termini	%	Count	Termini	%	Count
GT-AG	74.207	54669	GT-AG	70.626	16477
AT-AC	21.778	16044	AT-AC	25.302	5903
AT-AA	1.117	823	AT-AA	2.958	690
AT-AT	0.952	701	GT-GG	0.493	115
AT-AG	0.893	658	AT-AG	0.27	63
GT-AT	0.433	319	GT-AT	0.081	19
GT-GG	0.216	159	GT-TG	0.064	15
GC-AG	0.167	123	GC-AG	0.056	13
GT-CG	0.062	46	TT-AG	0.051	12
GT-TG	0.039	29	AT-CA	0.026	6
CT-AC	0.033	24	GT-CG	0.021	5
GG-AG	0.029	21	AT-AT	0.017	4
GA-AG	0.019	14	AT-TT	0.009	2
TT-AG	0.014	10	AT-CG	0.004	1
GT-TA	0.004	3	GT-AA	0.004	1
GT-CA	0.004	3	AT-GC	0.004	1
AT-GA	0.004	3	GT-AC	0.004	1
AT-GC	0.004	3	AT-GA	0.004	1
CT-AG	0.003	2	AT-GT	0.004	1
AT-CG	0.003	2			
CT-AT	0.003	2			
AT-CC	0.003	2			
AT-GT	0.003	2			
GT-AA	0.001	1			
AT-CT	0.001	1			
GT-AC	0.001	1			
AT-TT	0.001	1			
GT-TT	0.001	1			
GT-GA	0.001	1			
AT-TC	0.001	1			
AT-GG	0.001	1			
AC-AC	0.001	1			

Table S1. Minor intron termini from conserved intron clusters in animals and plants.

GO term	Hs-Ri	HsMIGs w/RiO	E	O/U	FE	FDR
intracellular transport (GO:0046907)	434	54	29.65	+	1.82	4.27E-02
small molecule metabolic process (GO:0044281)	549	12	37.51	-	.32	1.67E-03
carboxylic acid metabolic process (GO:0019752)	309	2	21.11	-	.09	9.13E-04
oxoacid metabolic process (GO:0043436)	314	2	21.45	-	.09	8.72E-04
organic acid metabolic process (GO:0006082)	319	2	21.79	-	.09	1.12E-03

Table S2. GO term enrichment for human MIGs with *Rhizophagus* orthologs (HsMIGS w/RiO), compared to all human-*Rhizophagus* orthologs (Hs-Ri). E: expected, O/U: over/under, FE: fold enrichment, FDR: false-discovery rate.
